# Impact of the Regulators *SigB*, *Rot*, *SarA* and *sarS* on the Toxic Shock *Tst* Promoter and TSST-1 Expression in *Staphylococcus aureus*

**DOI:** 10.1371/journal.pone.0135579

**Published:** 2015-08-14

**Authors:** Diego O. Andrey, Ambre Jousselin, Maite Villanueva, Adriana Renzoni, Antoinette Monod, Christine Barras, Natalia Rodriguez, William L. Kelley

**Affiliations:** 1 Service of Infectious Diseases, University Hospital and Medical School of Geneva, 4 rue Gabrielle-Perret-Gentil, CH-1211 Geneva 14, Switzerland; 2 Department of Microbiology and Molecular Medicine, University Hospital and Medical School of Geneva, 1 rue Michel-Servet, CH-1211 Geneva, Switzerland; University of Iowa Carver College of Medicine, UNITED STATES

## Abstract

*Staphylococcus aureus* is an important pathogen manifesting virulence through diverse disease forms, ranging from acute skin infections to life-threatening bacteremia or systemic toxic shock syndromes. In the latter case, the prototypical superantigen is TSST-1 (Toxic Shock Syndrome Toxin 1), encoded by *tst(H)*, and carried on a mobile genetic element that is not present in all *S*. *aureus* strains. Transcriptional regulation of *tst* is only partially understood. In this study, we dissected the role of *sarA*, *sarS* (*sarH1*), RNAIII, *rot*, and the alternative stress sigma factor *sigB* (σ^B^). By examining *tst* promoter regulation predominantly in the context of its native sequence within the SaPI1 pathogenicity island of strain RN4282, we discovered that σ^B^ emerged as a particularly important *tst* regulator. We did not detect a consensus σ^B^ site within the *tst* promoter, and thus the effect of σ^B^ is likely indirect. We found that σ^B^ strongly repressed the expression of the toxin via at least two distinct regulatory pathways dependent upon *sarA* and *agr*. Furthermore *rot*, a member of SarA family, was shown to repress *tst* expression when overexpressed, although its deletion had no consistent measurable effect. We could not find any detectable effect of *sarS*, either by deletion or overexpression, suggesting that this regulator plays a minimal role in TSST-1 expression except when combined with disruption of *sarA*. Collectively, our results extend our understanding of complex multifactorial regulation of *tst*, revealing several layers of negative regulation. In addition to environmental stimuli thought to impact TSST-1 production, these findings support a model whereby sporadic mutation in a few key negative regulators can profoundly affect and enhance TSST-1 expression.

## Introduction

*Staphylococcus aureus* is a versatile commensal human pathogen capable of causing a large spectrum of diseases ranging from skin infections such as furuncles and impetigo to severe systemic illness including bacteriemia, endocarditis, osteomyelitis, and deep tissue abscesses [1]. *S*. *aureus* can transiently colonize the anterior nares, axillae, perineum or the vagina in an estimated 30% of the world’s population without provoking any disease in the vast majority of cases [2]. Under certain circumstances, however, *S*. *aureus* can gain access to underlying tissue and potentially spread throughout the body, thus becoming a particularly dangerous opportunistic pathogen [1].

In order to provoke the wide range of disease pathology, *S*. *aureus* uses an arsenal of core-chromosomally encoded virulence factors (VF) including surface associated proteins, exoenzymes, and capsular polysaccharides, allowing adhesion, immune response evasion and tissue destruction [3]. The acquisition of mobile genetic elements often encoding one or more toxins, additional immune evasion factors, or antibiotic resistance determinants augments the VF repertoire and the potential disease spectrum [4–7].

Toxic shock syndrome (TSS) is a potentially fatal illness, characterized by fever, skin rash, desquamation, hypotension, and hemodynamic shock. The TSS Toxin-1 (TSST-1) is the causative toxin for the majority of menstrual-related and half of surgical-related TSS cases while the remaining cases are triggered by enterotoxins with superantigenic properties [8, 9].

The TSST-1 superantigen toxin, encoded by *tst (tstH)*, is not ubiquitous. It is found on various mobile pathogenicity islands (SaPI): SaPI1 (in strain RN4282), the closely related SaPIn1 (in strain N315), SaPIm1 (in strain Mu50), SaPI2 (in strain RN3984) and SaPIbov1 (in strain RF122) [10–13]. Estimates of the prevalence of strains encoding *tst* and sampled from healthy carriers ranges from 13–25%, indicating a large disease potential, yet the case incidence is relatively low (1-4/100,000) [14–18]. Recent studies demonstrate that SaPIs, including those encoding *tst*, can be packaged and efficiently disseminated by bacteriophage thus aiding their dissemination [10, 19]. Remarkably, *tst* is absent from most *S*. *aureus* model strains widely used for virulence regulation studies, such as NCTC8325 derivatives (RN6390, 8325–4, RN4220, SH1000, HG003), Newman, COL, and USA300, and thus the molecular pathways influencing this major superantigenic toxin remain largely unexplored [20, 21].

Various environmental triggers that influence the expression of TSST-1 have been described, such as glucose (via the *ccpA* catabolite repressor), O_2_ (possibly via the *srrAB* two-component system), magnesium ions, the α and β chains of hemoglobin, growth rate, pH, and TSST-1 itself [22–30]. Sub-inhibitory concentrations of nafcillin were found to induce TSST-1 expression at the transcriptional level whereas clindamycin, linezolid, and tigecycline were found to reduce TSST-1 expression [31]. A diverse number of chemical compounds displaying surfactant, membrane-active, or metabolic inhibitors also reduced TSST-1 expression [32]. The relatively high percentage of *S*. *aureus* circulating strains carrying *tst* coupled with the relatively low incidence of TSS argues strongly that *tst* expression sufficient to evoke disease occurs sporadically only with the proper combination of environmental and genetic regulation. Notably, this includes toxin susceptibility correlated with certain HLA Class II haplotypes, attenuated quorum sensing by probiotic strains within the vaginal mucosal microenvironment, circulating antibody titers sufficient to contain low level expression, as well as unexplored factors that lead to a reported 170-fold variation of TSST-1 levels detected in clinical *tst* samples [14, 33–35].

The complexity of *S*. *aureus* virulence regulation arises from the large number of global regulators involved in the process [28, 36, 37]. Two prominent factors thought to control TSST-1 expression are *agr* and *sarA*. The *agr* system, which responds to the quorum sensing auto-inducing peptide (AIP) and its effector trans-acting RNA termed RNAIII up-regulates TSST-1 [28, 38–40]. Whether the *agr* system effect depends upon direct interaction of RNAIII with *tst* transcript or alternatively by RNAIII-mediated Rot (repressor of toxins) inactivation has not been established [41–43]. The global regulator SarA has been shown to influence *tst* expression directly via binding of SarA to *sarA* cis-responding elements present on the *tst* promoter [39, 40]. The CcpA repressor responding to glucose binds to a cognate *cre* element overlapping the *tst* translation start site [25, 40]. Recent data show that CcpA DNA binding can also be regulated by phosphorylation mediated by HprK/HPr and metabolic cues as well as via the Stk1/Stp1 serine-threonine kinase implicated in cell wall stress sensing and antibiotic resistance [44, 45].

Previous knowledge regarding *tst* transcriptional regulation is derived from different strains and genetic strategies generating difficulties with the elaboration of a unified regulation pattern for this toxin. The various models include a P*tst*::*luxAB* transcriptional fusion reporter stably inserted in various *tst-* strains, overexpressing TSST-1 using *tst* cloned on multicopy plasmids, antisense knockdown, or clinical *tst+* strains harboring SaPIs such as RN4282 and MN8 [20, 22, 25, 26, 28, 30, 40, 46].

For the study reported herein, we primarily focused on RN4282, a prototypical strain bearing SaPI1, since this strain was used in the context of TSST-1 gene discovery and description of TSST-1 auto-regulatory properties and is amenable to genetic manipulation [11, 13, 26]. We discovered that the alternative stress sigma factor, *sigB* was required to exert strong repression of *tst* and TSST-1 expression. We propose that at least two different pathways mediate this effect through regulation of both *sarA* and *agr*/RNAIII. In addition, we found that *sarS*, a member of the SarA superfamily, imparts an additional level of negative regulation over *tst* expression but only consistently when combined with disruption of *sarA*. Collectively, these results provide additional insight into *tst* expression by defining negative regulators and thus exposing the potential for strongly enhanced TSST-1 expression in the event of sporadic mutation of these systems in circulating *S*. *aureus tst* ^+^ strains.

## Materials and Methods

### Bacterial strains

Strains and plasmids used in this study are listed in Table 1. *Escherichia coli* strains were grown in Luria-Bertani broth (LB) and *Staphylococcus aureus* strains were grown in Muller-Hinton broth (MHB). Media were supplemented with ampicillin (100 μg/ml), kanamycin (40 μg/ml), tetracycline (1–3 μg/ml), erythromycin (5 μg/ml) or chloramphenicol (15 μg/ml) when appropriate. Recombinant lysostaphin was obtained from AMBI Products LLC (Lawrence, New York). Derivatives of RN4282 containing the various indicated mutations were obtained by bacteriophage-mediated transduction using phage 80α and standard genetic procedures. All strain constructions were verified by PCR assay and appropriate primers.

**Table 1:** Bacterial strains and plasmids used in this study.

Strain/plasmid	Revelant genotype or characteristic	Source/reference
***E*.*coli***		
DH5α	restriction deficient DNA cloning strain	Gibco/BRL
***S*. *aureus***		
RN4220	restriction defective strain which accepts foreign DNA	[13]
N315P	MRSA strain N315 lacking penicillinase plasmid	[108]
RN4282	clinical strain harboring SaPI1 with *tst*	[11]
ALC1001	*sigB* mutant (Tn917) of RN6390	[99]
ALC2057	RN6390 *sarA*::*kan*	[63]
SH1000	8325–4 derivative with *rsbU* deletion repared	[21]
PC1072	8325–4 p_*tst*_ *-luxAB*::*geh* Tc^r^	[22]
DA101	PC1072 *sarA*::*kan*	[40]
KT201	8325–4 *sarH1(sarS)*::*pKT200* erm^r^	[59]
PM466	RN6390 *agr*-null, *rot*::*tet* Tc^r^	[64]
HI2672	*rot*::*ery* identical to WA525	[49]
WA400	8325–4 Δ*RNAIII-hld region*::*cat*	[109]
DA140	RN4282 *sigB-* ery^r^, 80α transductant of ALC1001	This study
DA141	DA140 + pDA205	This study
DA142	RN4282 *sarA*::*kan*, 80α transductant of ALC2057	This study
DA143	RN4282 *sarA*::*kan*, 80α 2^nd^ transductant of ALC2057	This study
DA150	SH1000 p_*tst*_-*luxAB*::*geh* Tc^r^, 80α transductant from PC1072	This study
DA155	RN4282 *sarS*::*pKT200*, 80α transductant from KT201	This study
DA156	DA142 *sarS*::*pKT200*, 80α transductant from KT201	This study
DA158	RN4282 Δ*RNAIII-hld region*::*cat*,80α transductant from WA400	This study
DA160	DA158 *rot*::*tet*, 80α transductant from PM466Δ*rot*	This study
AJ1049	RN4282 *rot*::*ery*, 80α transductant of HI2672	This study
AJ1055	AJ1049 + pWA163	This study
AJ1060	DA142 + pMK4 (empty)	This study
AJ1062	DA142 + pAJ973	This study
AJ1056	DA155 + pMK4 (empty)	This study
AJ1058	DA155 + pAM1865	This study
***Plasmids***		
pMK4	*E*.*coli-S*.*aureus* shuttle plasmid, amp^r^ cam^r^	[110]
pWA163	pAS1 containing *rot* under control of the *xylA* promoter Tc^r^	[49]
pDA200	pMK4 containing Not1-Kpn *Hu* promoter region cam^r^	[40]
pDA205	pDA200 containing a KpnI-PstI *sigB* fragment cam^r^	This study
pAJ973	pMK4 containing *sarA* under control of its native promoter cam^r^	This study
pAM1865	pDA200 containing a KpnI-PstI s*arS* fragment cam^r^	This study

### Construction of a *sigB* expression vector

Expression of *sigB* gene under the control of the nucleoid protein p*Hu* promoter was constructed as follows. Briefly, a polymerase chain reaction (PCR) amplification of the *sigB* gene was performed by using N315 genomic DNA as template and primers sigBKpnRBSF and sigBPstR2 (see Table 2). After digestion, the PCR fragment was cloned into KpnI and PstI restriction sites of pDA200, a pMK4 derivative containing *S*. *aureus HU* promoter sequence [40, 47]. The resulting plasmid, pDA205, was sequence verified and electroporated into non-restrictive *S*. *aureus* strain RN4220 prior to transfer to DA140 strain (Δ*sigB*). Restoration of a functional σ^B^ in the resulting complemented strain, DA141, was confirmed by detection of yellow pigmentation and by transcriptional analysis of the exclusively SigB-dependent gene *asp23* [48].

**Table 2:** Primers and probes used in this study.

Name	Primer sequence (5'-3')
sigBKpnRBSF	GGGGTACCAGGAGGTGAATGTCTAATGGCGAAAGAGTCGAAATCAGC
sigBPstR2	AACTGCAGCTATTTATGTGCTGCTTCTTGTAATTTCTTAA
sarAXbaBamHI	TGGTCTAGAGGATCCGTGCCATTAGTGCAAAACCTCTTAACA
sarAHindIIIPst	TATAACGTTCTGCAGGCGTTGATTTGGGTAGTATGCTTTGAC
sarSKpnRBSF	CGGGGTACCAGGAGGTGAATGTCTAATGAAATATAATAACCATGACAAAATTAGAGA
sarSPstR	AAAACTGCAGTTATTCAAAAACAAGATGTAAATGATCTTTATCTG
tst-39F	CCCTTTGTTGCTTGCGACA
tst-119R	GCTTTTGCAGTTTTGATTATTTGATT
tst-59T	TCGCTACAGATTTTACCCCTGTTCCCTTATCA
lux-1578F	CCGTTAACCCACACGCG
lux-1637R	TGCTCGTCGCATTCACAAAT
lux-1596T	TCACTGAAGGCGGTCCTGCGC

Underlined regions represent restriction enzyme sequences

### Construction of a *sarA* expression vector

Expression of *sarA* under the control of its entire native promoter containing its three known transcription start sites was constructed as follows. Briefly, PCR amplification of a region encompassing P3-P2-P1*sarA* sequence was performed by using N315 genomic DNA as template and primers sarAXbaBamHI and sarAHindIIIPst (see Table 2). After digestion, the PCR fragment was cloned into BamHI and PstI restriction sites of pMK4. The resulting plasmid, pAJ973, was sequence verified and electroporated into non-restrictive *S*. *aureus* strain RN4220 prior to transfer to DA142 strain, resulting in *sarA* restored strain AJ1062.

### Generation and complementation of *rot* mutation

Whereas certain experiments used *rot*::*tet* (PM466),we engineered an alternative *rot*::*ery* mutation in RN4282 by transduction from strain HI2672 (identical to WA525, kindly provided by D. Frees, Copenhagen, Denmark), generating AJ1049 strain (see Table 1) and compatible with the tetracycline resistant and xylose-inducible rot expression vector pWA163. pWA163 was electroporated into AJ1049, resulting in the conditional *rot* restored AJ1055 strain. Induction of Rot expression was obtained following previously published procedures [49].

### Total RNA extraction and real-time qRT-PCR assays

Overnight bacterial cultures were diluted in MHB (1/100) and grown at 37°C with vigorous agitation (210 rpm) and aerobic conditions (culture volume to tube ratio never exceeded 1:5 and with loosened screw caps) until OD_600_ = 1.5 to 2. Pilot studies determined by growth curves showed that sampling conditions corresponded with late exponential/post-exponential phase unless otherwise noted. Bacteria were harvested and RNA extracted as previously described [50]. The absence of contaminating DNA was always verified for every experiment by PCR using qRT-PCR probes in the absence of reverse transcription, as described [51]. *Tst*, *lux* and *sarS* qRT-PCR primers and probes were designed using Primer Express software and are indicated in Table 2. The *sarA* transcript levels were monitored using probe sets *sarA* 17F, 167R, and 45T, and RNAIII transcript levels were monitored with RNAIII 367F, 436R, and 388T as previously described [52]. The raw mRNA levels determined from the midpoint cycle threshold (*c*_*t*_) from the various strains were normalized to 16S rRNA levels, which were assayed in each round of qRT-PCR as internal controls. Data were collected for a minimum of three independent determinations. The statistical significance of strain-specific differences in normalized cycle threshold (*c*_*t*_) values of each transcript probe was evaluated by Student’s paired *t* test, and data were considered significant when *P* was < 0.05. For convenience in figure presentation, data were plotted to reflect fold change or %mRNA levels. Nevertheless, the reported *p* values always correspond to calculations with the normalized cycle thresholds. Normalized cycle threshold values for three independent experiments for the data displayed in each figure are provided in supplementary materials (S2 Fig and S1 Table).

### Immunoblot analysis

Culture supernatants of RN4282 strain and derivatives were collected at OD_600_ 1.5 to 2, and normalized based on OD_600_ values. Two concentration methods were found comparable. Equal amounts (20μg) of purified carbonic anhydrase gel chromatography standard (29kD) protein (Sigma) were added to each normalized supernatant as an internal control for sample recovery from spin microconcentrators (Millipore 10K MWCO), or trichloroacetic acid precipitation. The carbonic anhydrase spike also served as a marker for western transfer (followed by Ponceau Red staining of PVDF membranes as a loading control) since secreted proteins are often invisible by Coomassie staining, or can vary with strains. Alternatively after centrifugation, supernatants were precipitated with 10% (v/v) trichloroacetic acid (TCA), followed by one cycle of -20°C freezing and thawing, followed by centrifugation at 13000 rpm for 15 min. The final pellet was washed twice with 10% TCA and 80% acetone and resuspended in 40 μl of Tris-EDTA buffer, pH 8.0. Samples were spin concentrated according to the manufacturer’s recommendations. Aliquots of total exoproteins (8 μl) were loaded with an equal volume of Laemmli buffer on 12% SDS-PAGE gels and subsequently transferred onto a polyvinylidene difluoride membrane (PVDF, Bio-Rad). Uniformity of protein loading was confirmed post-transfer by Ponceau-red staining (S1 Fig). After blocking the membranes with 5% low fat milk in phosphate buffered saline, TSST-1 was probed with a 1:10,000 dilution of polyclonal anti-TSST-1 antibody (Thermo Scientific, Illinois, USA) followed by incubation with a secondary HRP-conjugated goat anti-rabbit antibody (1:10,000 BioRad). Chemiluminescence was detected using the Western Pico Super Signal reagent and the manufacturer’s recommendations (Pierce).

For time course experiments, cultures were diluted and grown as above and aliquots were removed at the indicated times and concentrated as above. For meaningful comparison, OD_600_ normalizations were performed for each time point, so that time points are comparable for each strain tested.

### Luciferase assay

Bacterial cultures of PC1072 and DA150 *luxAB* reporter strains were first grown overnight in MHB containing the appropriate antibiotic and then diluted in 5ml of antibiotic-free MHB, to a final OD_600_ of 0.01. Cultures were then grown in aerobic conditions as described above. For the assay of luciferase activity, bacterial cultures were first normalized to an OD_600_ of 0.5 in a total volume of 1 ml and immediately measured with a Glomax luminometer (Promega) by addition of 20 μl of 1% decanal solution (Sigma, freshly prepared (v/v) in absolute ethanol). The statistical significance of strain-specific differences in light emission (expressed in arbitrary units) was evaluated by Student’s paired *t* test, and data were considered significant when *P* was <0.05.

## Results

Many strains derived from NCTC 8325 are now known to harbor an 11 bp deletion in the gene encoding *rsbU* [53]. Consequently, the alternative stress sigma factor σ^B^ response remains greatly attenuated because σ^B^ remains complexed with its anti-sigma factor RsbW. This finding has prompted a re-evaluation of virulence factor regulation using strains possessing a functionally restored *rsbU* (for example, SH1000 or HG003) [21, 54]. In order to examine the effects of *sigB* disruption on *tst* transcription we made use of two distinct model systems: a P*tst*::*luxAB* transcriptional fusion using an approximately 400 bp region harboring the presumptive integrality of the P*tst* promoter as well as direct examination of *tst* transcription using strain RN4282 containing SaPI1 and *tst* in a native pathogenicity island genomic context [22, 40].

### SigB helps repress *tst* expression

**Fig 1:**
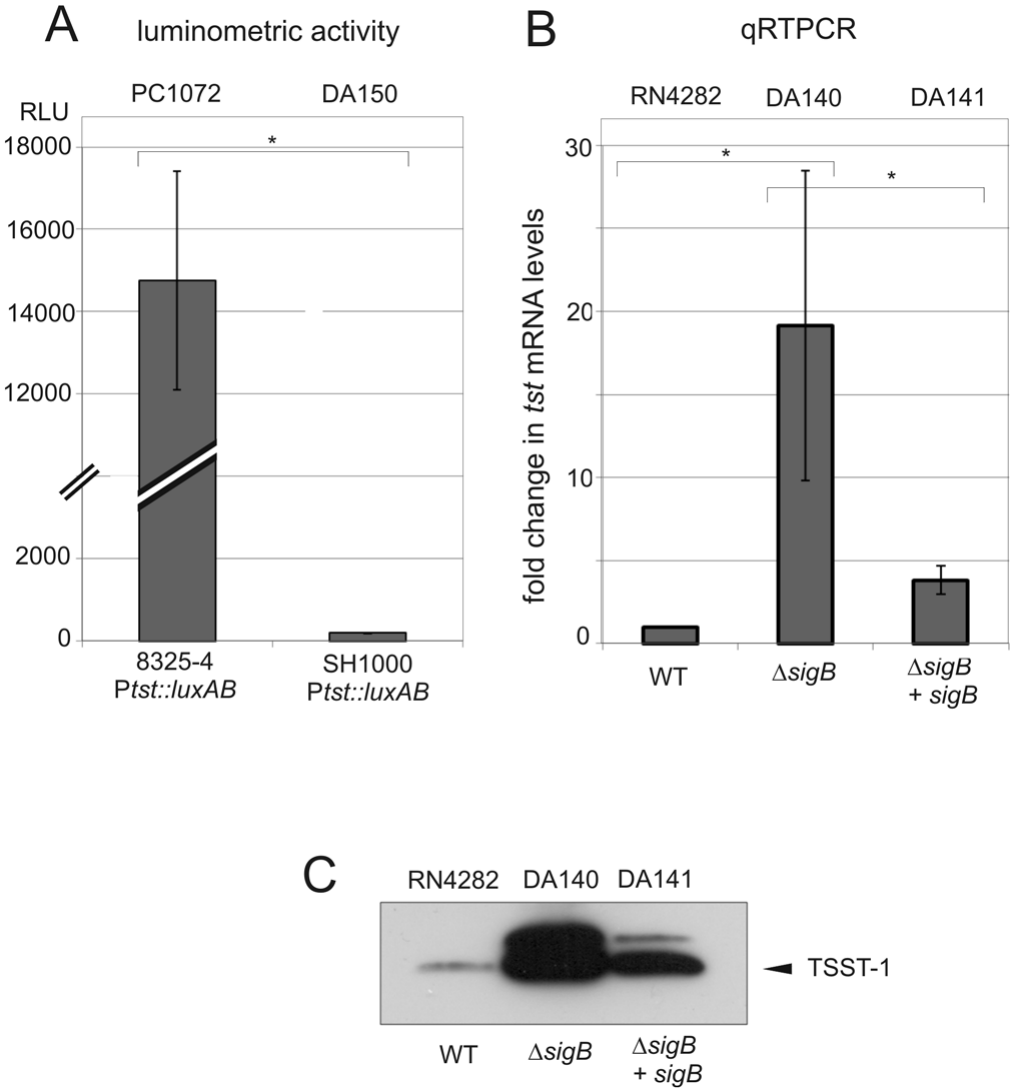
The effect of *rsbU* or *sigB* disruption on *tst* expression. **A.** Luciferase reporter assay for the *tst* promoter. The histogram shows measured luminometric activity of the indicated strains in post-exponential growth phase (Materials and Methods). RLU: relative light units. **B.** Quantitative qRT-PCR measurements of *tst* transcripts levels in *sigB* wt, *sigB* mutant, and *sigB* complemented strains, in post-exponential growth phase, setting reference RN4282 as 1. Bars show +/- standard deviations. All data were compiled from three independent experiments. Statistical significance was evaluated by Student’s paired *t* test, and data were considered significant when *P* was <0.05 **C.** Western blot of TSST-1, using anti-TSST-1 polyclonal antibody after precipitation from supernatants of the indicated strains. Note the appearance of a strong band corresponding to unprocessed precursor TSST-1 detected in the absence of *sigB* and diminished upon reintroduction of multicopy *sigB* (upper band). The experiment shown is representative of several independent experiments.

Previous studies performed in our laboratory showed strong activation of the *tst* promoter in post-exponential growth phase using a P*tst*::*luxAB* transcriptional fusion present in the *rsbU*- strain PC1072 [39, 40]. Since σ^B^ activity in *rsbU*- strains is markedly lower than *rsbU+* strains, we hypothesized that σ^B^ activity could conceivably affect *tst* promoter activity [55]. To address this point, we first analyzed the *tst* promoter activity in *rsbU-* and *rsbU*+ strain backgrounds (8325–4 and SH1000 derivatives, respectively). The results are shown in Fig 1A.

We observed that luciferase activity levels in the *rsbU*+ strain (DA150) were significantly lower compared to the luciferase activity recorded from the *rsbU-* PC1072 strain. Several independent isolates of DA150 arising from the bacteriophage-mediated transduction transfer of the p*tst-luxAB* reporter showed similar results suggesting that the observed reduction of the luciferase reporter activity was linked with the restoration of a functional σ^B^ pathway and did not arise from a trivial spurious mutation. To further explore the role of *sigB* on *tst* expression, we next examined *tst* transcription directly by performing qRT-PCR and using strain RN4282 and its corresponding isogenic Δ*sigB* derivative DA140. In post-exponential growth phase, the steady-state *tst* transcripts levels were strongly and significantly increased by at least an order of magnitude in the Δ*sigB* strain DA140 compared to the parent strain RN4282 (Fig 1B). A similar induction (approximately 10-fold) was observed in exponential growth phase prior to the onset of *agr*-mediated quorum sensing regulation of *tst* via RNAIII production [40]. Reintroduction of *sigB+* cloned on a multicopy plasmid, DA141, significantly reduced *tst* transcript levels compared to the Δ*sigB* mutant (Fig 1B). Taken together, we conclude from these studies that disruption of the alternative stress sigma factor σ^B^ pathway by either of two methods, *sigB* deletion, or by use of defective *rsbU*, results in significantly enhanced *tst* transcription.

In order to extend these findings, we next examined the impact of *sigB* disruption on secreted extracellular TSST-1 protein levels. Culture supernatants of RN4282 and its mutant derivatives were sampled in the post-exponential growth phase under the same conditions as for RNA analysis and examined by western blot analysis using anti-TSST-1 antibody. The results are shown in Fig 1C.

Strikingly, we observed higher amounts of TSST-1 in supernatants prepared from the Δ*sigB* strain compared to supernatants obtained from either RN4282 or to the *sigB+* restored strain DA141. Re-introduction of *sigB*+ on a multicopy plasmid DA141 led to a significant diminution of TSST-1 in culture supernatants as judged by western blot analysis. The results showed, however, that σ^B^-dependent repression was not restored entirely to wild type levels observed in RN4282 and this may be explained by experimental system employed and use of a multicopy plasmid. Nevertheless, these results point to a strong concordance between the *tst* transcriptional profile and the secreted TSST-1 profile (Fig 1B and 1C) with regard to *sigB*. Collectively, we conclude from these results that the presence of a functional *sigB* exerts a strong repressive effect on *tst* and subsequent TSST-1 expression in the RN4282 genetic background.

### Expression of the global virulence regulators *sarA*, RNAIII and *sarS* in RN4282 and its Δ*sigB* derivative

**Fig 2:**
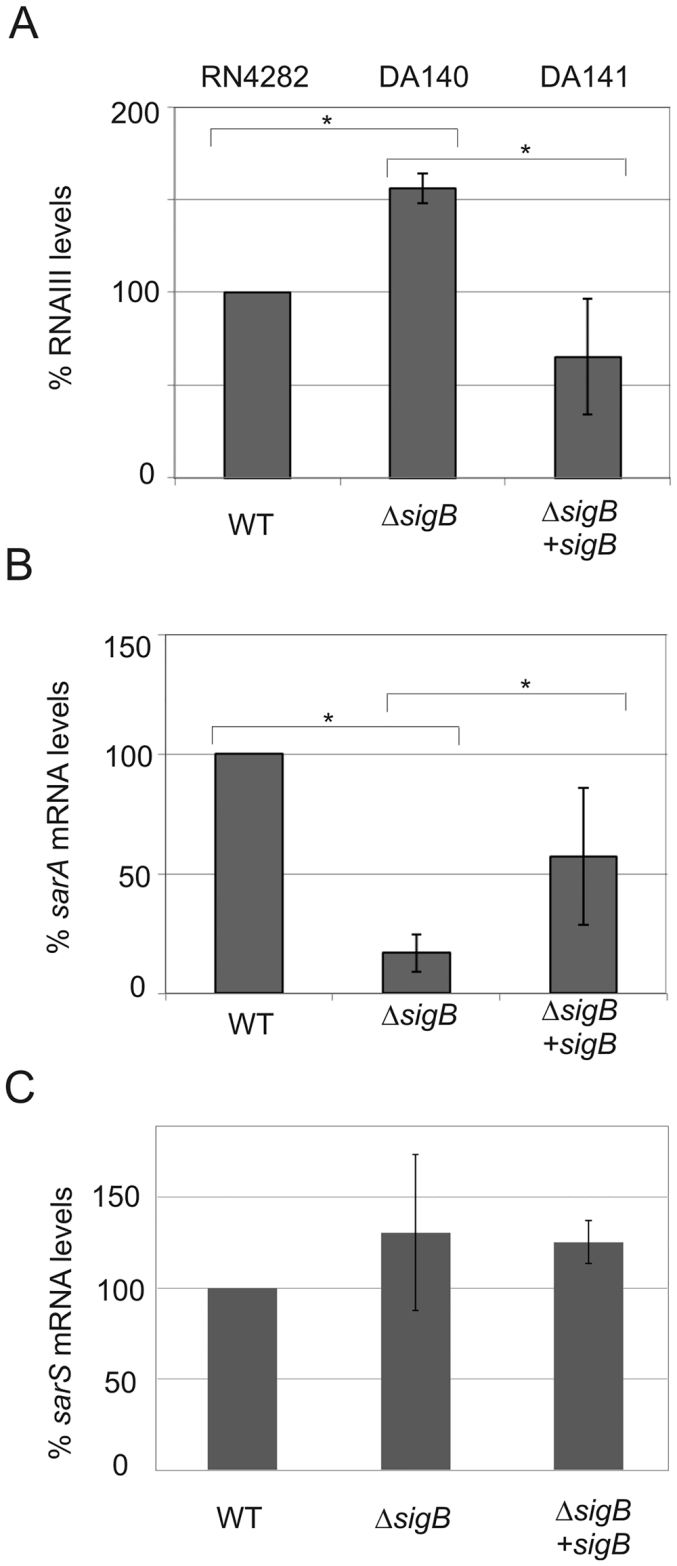
The effect of *sigB* disruption on RNAIII (A), *sarA* (B) and *sarS* (C) transcript levels in RN4282 and its derivatives using quantitative qRT-PCR measurements of RNA expression. Relative changes are shown in each panel using RN4282 as 100%. Bars show +/- standard deviations and all data were compiled from three independent experiments. Statistical significance was evaluated by Student’s paired *t* test, and data were considered significant when *P* was <0.05.

Inspection of the *tst* promoter sequence failed to reveal the presence of a canonical σ^B^ consensus recognition sequence (GTTTWWN_12-15_ GGGWAW), previously established in *Bacillus subtilis* and refined following *S*. *aureus* transcriptomic analysis of a Δ*sigB* mutant [48, 56, 57]. Our previous work had detected only a single *tst* transcription start site by 5’-RACE analysis in RN4282 [40]. Since we found no σ^B^ consensus sequence within the *tst* promoter, we examined the possibility that an indirect regulatory mechanism could account for the *sigB*-mediated effect on TSST-1 expression. Since *sigB* has been previously shown to have an effect on *sarS*, *sarA* and RNAIII transcription, we measured steady state transcript levels in RN4282 and its isogenic Δ*sigB* derivative strain DA140 by qRT-PCR using probes specific for *sarS*, *sarA* and RNAIII (Fig 2) [48, 58, 59]. Interestingly, we found that the *sigB* disruption had no significant effect on *sarS* mRNA levels in this strain background (Fig 2C), while in contrast, a significant effect of the *sigB* disruption was observed on *sarA* (Fig 2B) and a modest effect on RNAIII (Fig 2A). RNAIII steady state levels in post-exponential phase revealed a moderate, yet statistically significant, increase in RNAIII level (150%, *p*<0.05) in the Δ*sigB* mutant compared to the parental RN4282 strain, or the *sigB+* restored strain DA141 (Fig 2A). However, whether this slight, but statistically significant, change in RNAIII levels is physiologically relevant is difficult to ascertain. We observed a five-fold reduction (20% of the wild type level) of *sarA* mRNA in the Δ*sigB* mutant strain compared to the parental RN4282 strain (Fig 2B). Restoration of *sigB* on the multicopy vector (strain DA141) did significantly increase *sarA* mRNA levels almost three-fold compared to *sigB* mutant, reaching 57% of wild-type levels. Interestingly, this incomplete rescue of *sarA* levels, observed in the *sigB* restored strain DA141, mirrors the incomplete restoration of *tst* repression in the same DA141 strain (Fig 1B). Taken together, these results show that *sigB*-dependent modulation of *tst* transcription most likely involves contribution including, but perhaps not restricted to, a molecular pathway involving SarA regulator and RNAIII.

The consequences of disruption of *sigB* for virulence factor regulation have consistently noted reciprocal regulation; notably, deletion of *sigB* leads to enhanced RNAIII transcription and decreased SarA transcription [48]. The regulation of *tst* was not reported in that study, but our results above further confirm this reciprocal regulation. To explore regulatory circuitry further, we next examined the roles of RNAIII, *rot*, *sarA*, and *sarS*.

### Effect of the virulence regulators RNAIII and *rot* on TSST-1 expression in RN4282

**Fig 3:**
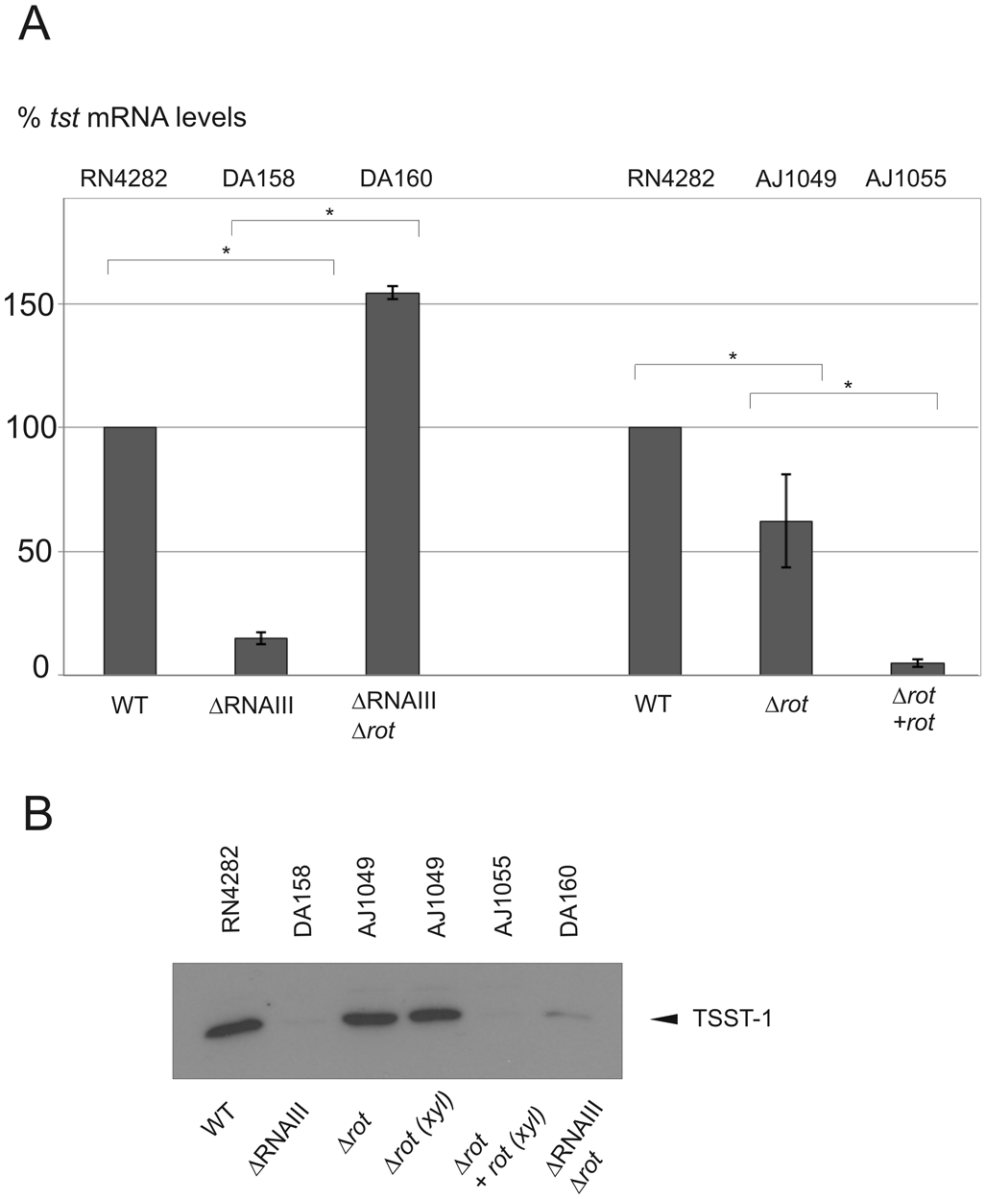
The effect of RNAIII and *rot* disruption or overexpression on *tst* and TSST-1 in RN4282. **A.** Quantitative qRT-PCR measurements of *tst* expression and setting RN4282 as 100%. Bars show +/- standard deviations. All data were compiled from three independent experiments. Statistical significance was evaluated by Student’s paired *t* test, and data were considered significant when *P* was <0.05. **B.** Western blot of TSST-1, using anti-TSST-1 polyclonal antibody after precipitation from supernatants of the indicated strains. In lane 4 and 5 both samples include xylose to discard effect of the latter on *tst* expression. The experiment shown is representative of several independent experiments.

The quorum sensing two component system *agr* modulates the expression of RNAIII, which in turn can affect gene expression by various mechanisms [43]. One of the genes translationally regulated by RNAIII is *rot* (repressor of toxins), which encodes a DNA binding protein thought to act as a global repressor of virulence genes including, for example, hemolytic toxins [41, 60, 61]. Rot is thought to act primarily as a repressor, although there are reported exceptions and recent work suggests that Rot could also cooperate positively with the two-component system SaeRS to activate expression of superantigen-like exoproteins [42, 62]. To address the question whether *rot* positively or negatively regulated *tst* expression, we constructed isogenic derivatives of RN4282 lacking RNAIII (DA158), *rot* (AJ1049) and both RNAIII and *rot* (DA160); complementation of the *rot* deletion by its expression on a xylose-inducible vector, was also analyzed (AJ1055). The results are shown in Fig 3.

We observed that loss of RNAIII (strain DA158) resulted in a significant decrease of *tst* transcription (>5-fold) and expression, consistent with our observations that *tst* transcripts are virtually undetectable in exponential growth prior to the onset of *agr*-mediated sensing system. Disruption of *rot* provoked a slight, but significant diminution of *tst* transcripts levels (Fig 3A), without a consistently visible change on TSST-1 secreted levels (Fig 3B). The restoration of Rot expression from a multi-copy complementing plasmid (AJ1055) strongly reduced both *tst* transcripts levels and TSST-1 secreted levels compared to the **Δ** *rot* strain (AJ1049); xylose alone in the absence of the complementing plasmid had no effect on *tst* expression. Taken together, these results suggest that RNAIII enhances TSST-1 expression in post-exponential phase as expected; whereas *rot* disruption has, under our experimental conditions, no consistently measurable effect at the toxin protein expression level, but when overexpressed strongly represses at both transcriptional and expression level. These results are consistent with the model whereby RNAIII/*agr* activates *tst* transcription. Of note, no Rot binding site consensus has been identified in *S*. *aureus* to date. Finally, combination of both RNAIII and *rot* mutations only slightly increased *tst* transcription (1.5-fold), but consistently did not affect TSST-1 expression, as judged by western blot analysis (Fig 3).

### Effect of the global virulence regulators *sarA* and *sarS* on TSST-1 expression in RN4282

We next examined the consequences of the disruption of the global regulator *sarA*, as well as the related SarA-family member *sarS* on *tst* expression in RN4282. Despite our findings above showing a lack of evidence for *sigB*-dependent modulation of *sarS*, we nevertheless chose to examine the consequence of *sarS* disruption on *tst* expression because previous study had demonstrated a key role for *sarS* in the regulation of various surface and secreted virulence factors (including alpha-toxin), together with the finding that *sarA* controls the expression of *sarS* through an intermediate step via another *sarA* family member, *sarT* [59, 63–65]. Sar-family members are thought to interact in a subtle and complex network to control the expression of both surface and secreted virulence factors [66]. Importantly, not all *S*. *aureus* strains encode *sarT* and *sarU*, which together are found on a small islet not considered part of the core *S*. *aureus* genome [67]. As a first step, we performed direct sequence analysis of RN4282 and confirmed the presence of the *sarT/sarU* encoding islet (data not shown).

**Fig 4:**
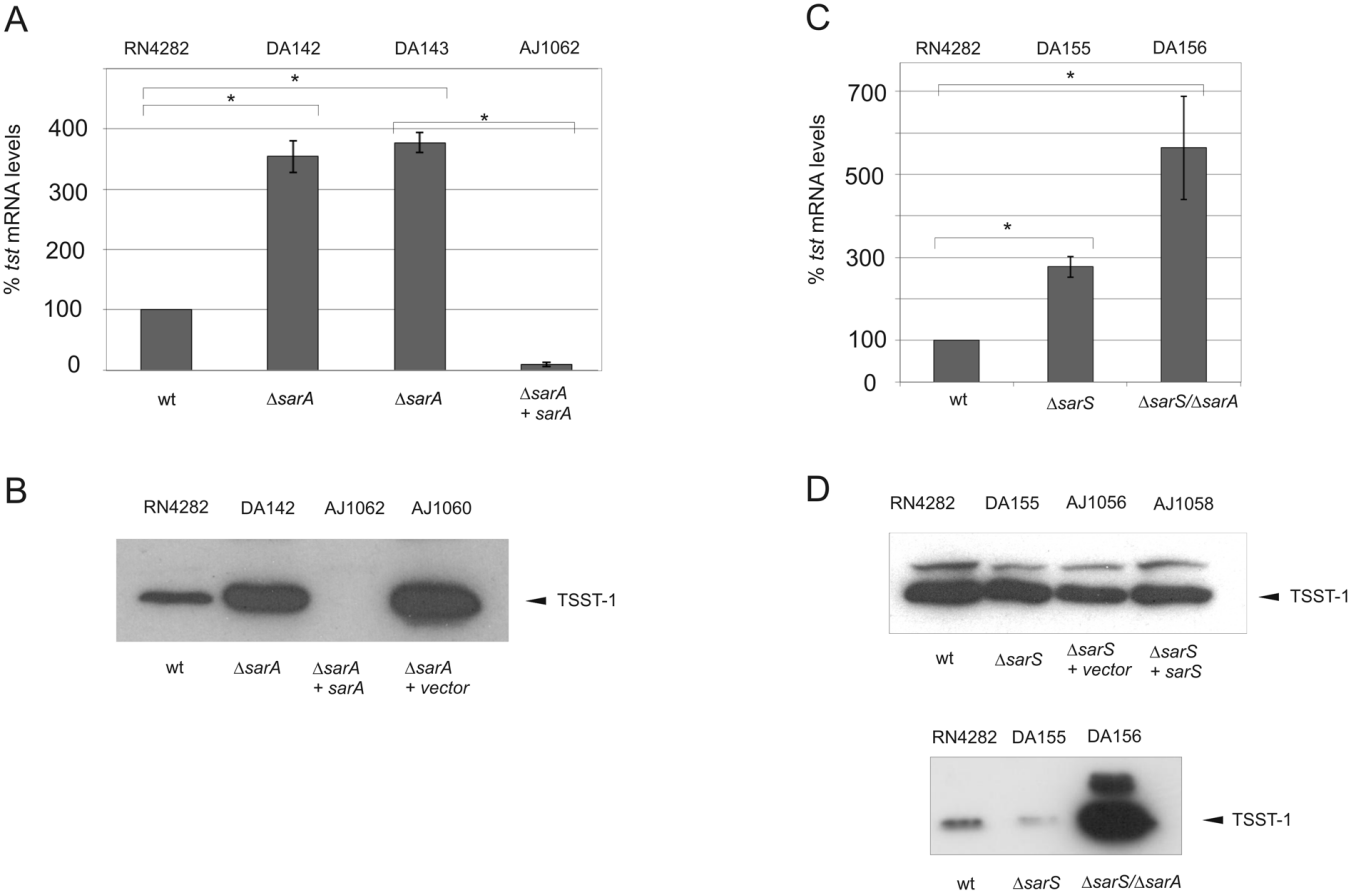
The effect of *sarA* and *sarS* on *tst* and TSST-1 toxin expression in RN4282. **A and C.** Quantitative qRT-PCR measurements of *tst* expression in the indicated strains, setting RN4282 as 100%. Bars show +/- standard deviations. All data were compiled from three independent experiments. Statistical significance was evaluated by Student’s paired *t* test, and data were considered significant when *P* was <0.05. **B and D.** Western blot of TSST-1, using anti-TSST-1 polyclonal antibody after precipitation from OD-normalized supernatants of the indicated strains (Materials and Methods). The experiment shown is representative of several independent experiments.

We observed that *tst* transcription was significantly increased (approximately 3.5-fold) in the *sarA* disruption strain DA142 compared to its isogenic parental strain RN4282 (Fig 4A). Importantly, this enhanced transcription was mirrored at the protein level, since western blot analysis detected markedly increased TSST-1 in normalized culture supernatants of DA142 compared with RN4282 supernatants (Fig 4B). Similar results were obtained using an independent *sarA* disruption, DA143. It is possible that western blots using supernatants from *sarA* disruption strains have enhanced proteolytic activity that influences detection of TSST-1 (leading to underestimation) since we detected reductions in the carbonic anhydrase control protein added to monitor supernatants only in *sarA* deletion strains (S1 Fig) [68].

In order to further confirm this repressive role of SarA we analyzed the effect of *sarA* complementation with a multi-copy pMK4 plasmid encoding *sarA* under control of its entire native P1/2/3 promoter upstream sequence. Complementation resulted in a strong repression of both *tst* transcripts and TSST-1 secreted toxin levels (Fig 4 panels A and B).

Collectively, we conclude that *sarA* functions as a negative regulator of *tst* in the RN4282 strain background. Moreover, these findings suggest that in the absence of σ^B^, a combination of higher stimulatory RNAIII and reduced SarA levels could conceivably account for the strong induction of *tst*.

We next prepared isogenic strains containing disruption of either *sarS* (DA155), both *sarA*/*sarS* (DA156), and complementation of the *sarS* deletion. *Tst* expression was compared between each strain set by both qRT-PCR and western blot analysis of TSST-1 in normalized culture supernatants. The results revealed that deletion of *sarS* gene (DA155) significantly increased *tst* transcript levels nearly 3-fold compared to RN4282 (Fig 4C), but this stimulatory effect was not mirrored by increased TSST-1 secreted toxin (Fig 4D). Indeed, we observed in multiple trials that TSST-1 levels obtained from DA155 supernatants were equivalent, or slightly reduced compared with RN4282, but not increased compared with RN4282. Finally, we also consistently observed that complementation of DA155 with plasmid-encoded *sarS* did not detectably influence TSST-1 levels (Fig 4D).

In contrast with the relatively minor role we detected for *sarS* disruption alone, the disruption of both *sarA*/*sarS* resulted in a significant 6-fold increase in *tst* expression compared with RN4282. This result is in contrast with the observed 3.5-fold increase in *tst* transcripts detected with the *sarA* disruption alone (Fig 4A) indicating that *sarS* disruption, nevertheless can, under certain conditions, influence *tst* expression. Western blot analysis showed that corresponding supernatants from the strain DA156 *sarA*/*sarS* double mutant resulted in strong production of TSST-1 compared to RN4282 (Fig 4D, lower panel). Western blots of DA156 supernatants also showed the appearance of a strong band corresponding most likely to pre-TSST-1 as we had observed with supernatants obtained from DA140 containing the *sigB* disruption (Fig 1C), but not from DA142 *sarA* disruption supernatants alone (Fig 4B). Collectively, these results led us to conclude that *sarS* exerts little, or no significant regulation upon *tst* transcription in RN4282 strain, at least in our experimental conditions. In addition a *sarA*-dependant negative regulation can be detected independently of the presence of *sarS*. SarS would thus not appear to play a significant role in governing repression of TSST-1 expression in this strain background alone, but *sarS* can exert a synergistic effect on TSST-1 expression when combined with disruption of *sarA*.

### Time course expression of TSST-1

**Fig 5:**
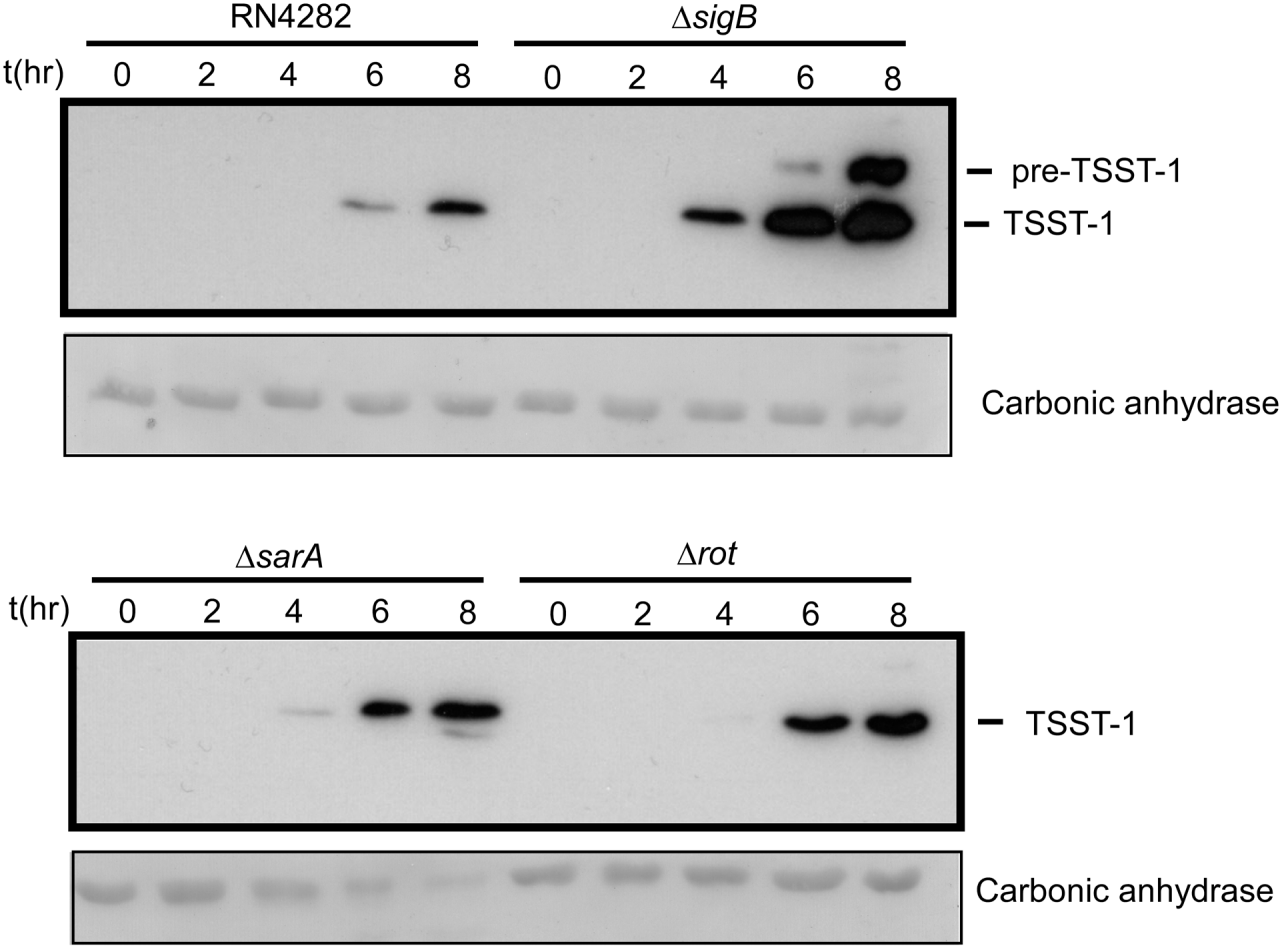
Western blot showing the time course production of TSST-1 in supernatants of the indicated wild type or mutant strains. Samples, at each time point, were OD_600_ normalized to each other. Prior to supernatant concentration, samples were spiked with a fixed amount of pure carbonic anhydrase as an internal preparation loading control, and is shown as the Ponceau stained band from the PVDF membrane (Materials and Methods). Note the strong production of TSST-1 from the Δ*sigB* strain (DA140).

Results presented above revealed that several of our mutants resulted in enhanced production of TSST-1 in post-exponential phase. We next wished to examine the possibility that various mutations resulted in detectable changes in the production of TSST-1 throughout the progression of exponential phase and early post-exponential phase as well. Time course experiments were performed using three deletion mutant strains Δ*sigB*, Δ*sarA* and Δ*rot*. TSST-1 protein levels were assessed by western blot analysis at two-hour time intervals following dilution of washed overnight cultures to remove residual TSST-1 (Materials and Methods). The results are shown in Fig 5.

Consistent with data presented above, the *sigB* deletion strain profoundly enhanced TSST-1 expression at 4, 6 and 8 hours of growth compared with wild type RN4282, and TSST-1 was clearly detected at 4 hours, whereas no TSST-1 was detected in supernatants from RN4282. A similar effect was observed with the *sarA* deletion, but TSST-1 levels were only moderately enhanced compared with RN4282; TSST-1 was clearly detected at the four hour time point, a condition where no TSST-1 was detected in RN4282 supernatants. Finally, the effect of *rot* deletion alone indicated a detectable enhancement of TSST-1 at 6 hours compared with RN4282, and a slight enhancement at 8 hours compared with RN4282. It is important to point out that culture supernatants in these time course experiments were OD normalized to each other at the indicated time points for the purposes of interstrain comparison.

## Discussion

In this study, we examined multiple regulators controlling *tst* transcription and TSST-1 expression. There is only scant knowledge about the regulation of this major virulence factor situated on a mobile genetic element and absent from most strains used for virulence regulation studies. Our present study now uncovers evidence for the negative regulation of *tst* expression by several factors including *sigB*, *sarA*, and probably *rot*, at least when significantly overexpressed. The remarkably strong production of TSST-1 detected in culture supernatants arising from disruption of either *sigB*, *sarA*, or disruption of *sarA* in combination with disruption of *sarS* underscores the multifactorial nature of *tst* regulation. We do not at this time fully understand the impact of various possible regulatory protein combinations upon the *tst* promoter.

**Fig 6:**
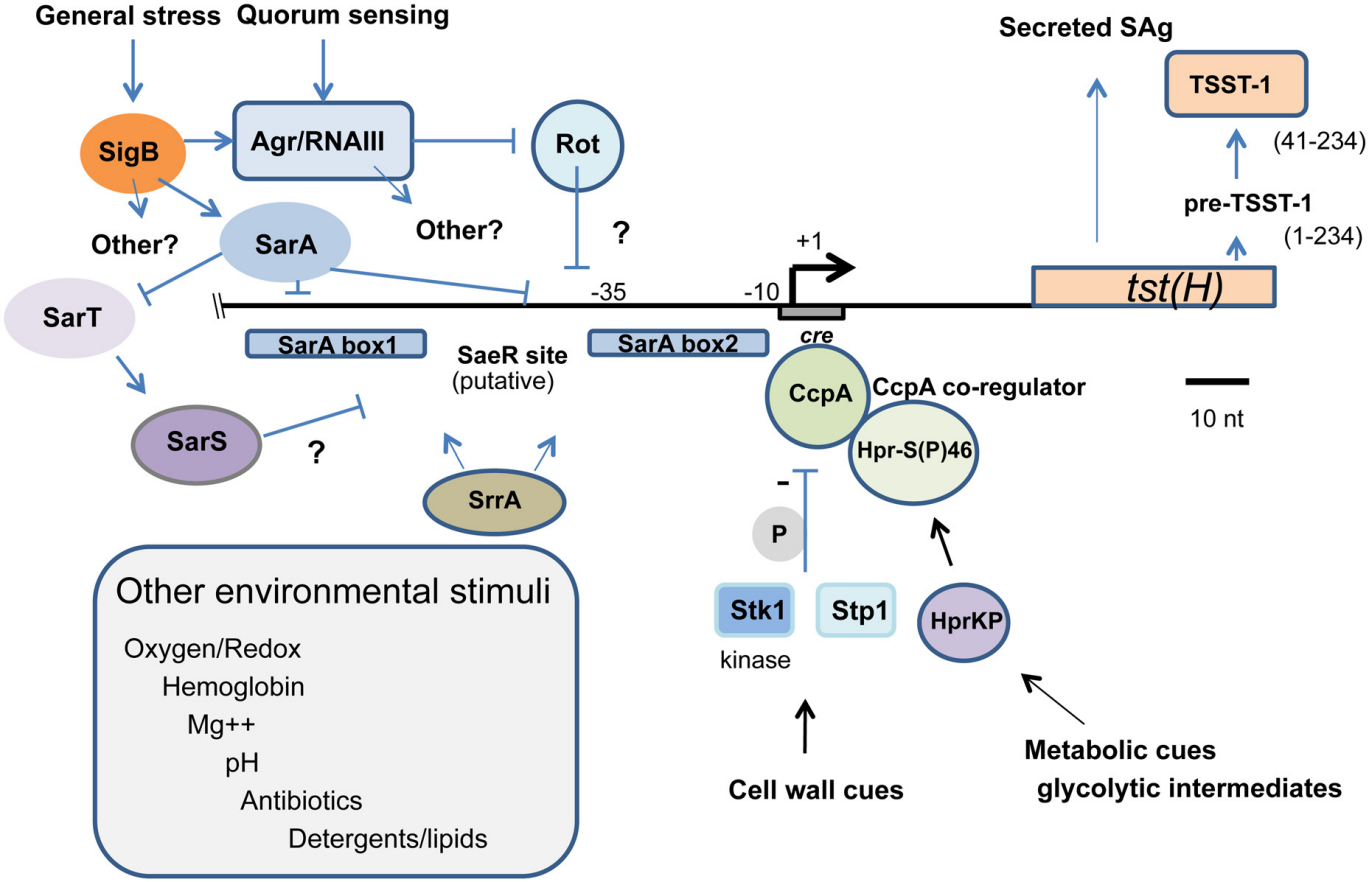
Model depicting the regulation of the TSST-1 superantigen in *S*. *aureus*. The horizontal line shows the position of the transcriptional start site (+1) determined in RN4282 and 37 nucleotides upstream of the translation start site [40]. The positions of SarA boxes 1 and 2 are shown and correspond to their positions determined by both SarA consensus and direct DNA binding assay [40]. Negative regulators determined in the study reported herein include **σ** ^B^, SarA, and Rot. SarA modulation of SarS is thought to occur via SarA negative regulation of the SarS activator SarT [65]. Catabolite control protein a (CcpA) binding to its cognate cis- acting *cre* site mediates additional *tst* repression by integrating signals from glycolytic intermediates via phosphorylation of the CcpA co-regulator as well as direct phosphorylation (*grey P*) via the Stk1 kinase which affects its DNA binding affinity [25, 44, 69]. The Stk1 S/T kinase and its cognate phosphatase, Stp1, may impart additional levels of control via the phosphorylation of SarA and the nucleoid protein, Hu, for example [45, 70, 71]. SrrA, the response regulator of the SrrAB two-component sensor, is thought to control *tst* regulation in response to oxygen and coenzyme Q [29, 30, 69, 100]. SrrA specific binding has been detected in the *tst* promoter region (Andrey, manuscript in preparation). DNA sequence with strong similarity to the consensus DNA binding site for the response regulator SaeR is shown. Although the precise involvement of SaeRS in *tst* regulation is unknown, it may help coordinate response to pH together with *sigB* [34, 101, 102]. Additional environmental stimuli known to affect TSST-1 expression are boxed although the precise genetic factors mediating these effects have yet to be defined [32, 102–107].

Our study findings lead us to propose that in addition to environmental cues that impact sensory systems governing *tst* expression, sporadic mutation leading to disruption of one or several regulators could conceivably have a profound impact on TSST-1 expression and perhaps shift production sufficiently to influence progression to overt toxin-mediated disease. Given that other factors such as catabolite control protein CcpA are known to negatively regulate *tst*, we integrate a number of these results in a model of *tst* regulation depicted in Fig 6 [25].

The model depicts the arrangement of known features of the *tst* promoter region previously described by our own work that precisely mapped two SarA binding sites, together with mapping of the *cis*-acting catabolite response element (*cre)* site mediating DNA-protein interaction with CcpA [25, 40]. Presumptive DNA binding sites for Rot and other regulators are presently unknown. Our model also depicts several additional levels of regulation, notably the modulation of CcpA DNA binding by Stk1 kinase dependent phosphorylation and association with its co-regulator that integrates metabolic cues by sensing glycolytic intermediates [44, 69]. Stk1 and its cognate phosphatase Stp1 constitute a serine/threonine kinase sensor system linked to virulence regulation and phosphorylation of other global regulators such as SarA, the nucleoid protein Hu, and MgrA [45, 70–72]. There are a number of environmental factors that have been shown or are suspected to impact *tst* expression for which there is presently no genetic explanation of the underlying sensory system and regulatory circuitry. The model provides a framework for further understanding the nature of environmental inputs known to modulate *tst* expression and the underlying genetic elements that integrate this regulation. Some of these points will be discussed in more detail below.

Our results showed that *sigB* helps to impart particularly strong repression of *tst* expression in both RN4282 and 8325–4 genetic backgrounds. Sequence inspection failed to reveal evidence for a σ^B^ consensus in the *tst* promoter, and thus σ^B^ most likely exerts its repressive effect indirectly. We presented evidence to support that indirect repression of TSST-1 expression by σ^B^ is mediated, in part, by the modulation of *sarA* and *agr/RNAIII* systems. To support this hypothesis we confirmed in RN4282 strain that *sigB* positively regulates *sarA* transcription and represses *agr/RNAIII*, in accordance with existing models where one of the three *sarA* promoters, *sar*P3, is controlled by σ^B^ [28, 73]. We also established that *agr/RNAIII* enhances TSST-1 expression and that *sarA* strongly represses it in RN4282. The additional effects of both upregulated *sarA* levels and low RNAIII levels thus likely participate in TSST-1 repression.

Previous detailed study of the *sigB* regulon has shown that it controls a vast network of more than 250 genes in *S*. *aureus* and that diverse environmental stress stimuli such as ethanol, and acid shock lead to σ^B^-dependent gene expression [48]. Despite a demonstrated role for *sigB*-dependent regulation of *agr/RNAIII* and *sarA* described above, we cannot, of course, exclude that other *sigB*-dependent factors are also involved in *tst* regulation. Inspection of the various fold-changes in transcription measured in our study indicated that loss of *sigB* was by far the most consequential compared with disruption of RNAIII, *rot*, or *sarA*. This finding strongly suggests that additional *sigB*-dependent regulators of *tst* await discovery. Recent studies have explored the indirect regulation of certain genes lacking a σ^B^ consensus via SpoVG and/or the two-component system ArlRS, for example [74–77]. In addition, small non-coding RNAs have been described that are controlled by *sigB*, for example, RcsA, RcsD, and RcsF, but to date, their precise regulatory role is unknown [43, 78, 79]. Recent work also suggests that pigment-deficient strains, possibly related to defects in *sigB*, arise during the course of *S*. *aureus* community development and strong bacterial competition [80]. Future studies will certainly shed new light on *sigB*-dependent virulence factor regulation.

SarA is clearly important for the transcriptional regulation of *tst*, and here we show that *sarA* is a potent repressor of TSST-1. These results are in contrast with previous published studies, however, where *sarA* was shown by ourselves and others to enhance *tst* promoter expression in an alternative strain background namely, 8325–4 using a *tst* promoter reporter (P*tst*::*luxAB* PC1072) strain; to our knowledge this effect was observed only in the *S*. *aureus* NCTC8325 genetic background and this may arise because of the *rsbU* and *sarS* defect in this strain [89] leading to defective *sigB* levels and concomitant alterations in regulatory protein combinations acting on the *tst* promoter [39, 40]. The disparate phenotypes of *sarA* mutation, depending upon the studied strain, has been previously reported regarding the control of several virulence factors, and particularly *hla* encoding hemolysin alpha exotoxin [28, 64, 81–84]. While *sarA* first appeared to be a repressor of exoproteins and an activator of membrane-bound proteins, when originally identified in a transposon Tn*917*LTV1 mutagenesis, several subsequent studies, primarily performed with NCTC8325 derivatives, showed *sarA* to be necessary for full production of secreted exoproteins, including α-hemolysin (*hla*) [81, 82, 85, 86]. Further studies showed that *sarA* repressed exoprotein synthesis in strains unrelated to NCTC8325 such as Newman and UAMS-1, confirming important differences in regulatory patterns among *S*. *aureus* strains [83]. The enhancing effect of SarA on virulence factors could not be found in other genetic backgrounds than NCTC8325. Notably little information was available regarding TSST-1 regulation in NCTC8325 related strains.

In this report we also have shown that *rot* can modulate *tst* transcription, conferring in RN4282 strain a potential additional layer of negative regulation on TSST-1. The absence of clear effect of *rot* disruption combined with the strong repression observed in multi-copy plasmid complementation conditions, suggests a role for gene dosage for this regulator and possibly depending upon access to the cis-acting *tst* promoter sequences and competition between regulators. Our data do not contradict the model that entry into post-exponential phase and activation of *agr* quorum sensing results in RNAIII-mediated inhibition of *rot* transcription and relief of Rot-dependent repression [61]. Recent work suggests, however, the possibility that by analogy with SarS, inter-strain differences and variation in Rot levels during growth phase may nevertheless contribute unpredictable levels of variation in Rot-dependent virulence factor regulation [87].

SarS is not a major regulator of *tst* in RN4282, at least in our experimental conditions. SarS was reported to be controlled positively by SarT; while SarT itself was found to be negatively regulated by SarA [65]. SarS-mediated repression of *tst* may be subject to strain-dependent variation for several reasons: *sarT* and *sarU* genes are not found on all *S*. *aureus* strains, *sarS* transcript levels are reduced in the widely used laboratory strain 8325 lineage, and *sarS* transcription and steady state protein levels may be growth phase dependent [67, 88–90].

How could SarA contribute to the regulation of *tst*? Specific SarA binding to the *tst* promoter has been determined and we have detected at least two sites by in vitro assay in our previous study [40]. Specific SarS binding has not been explored and a consensus site has not been defined for this regulator in *S*. *aureus*. Notably, however, recent work showed that SarA could bind and bend DNA at the *agr* P2 promoter, thereby promoting topological changes that allow additional regulatory protein-protein interactions at this promoter during exponential phase [91]. Interestingly, an additional Sar-family member, SarR, was shown in this study to bind to regions overlapping the SarA site and with higher affinity, but without inducing significant DNA bending. The consequence of this is thought to lead to a post-exponential reduction in *agrP2* promoter activity arising from displacement of SarA and loss of SarA-induced DNA bending. Viewed in this context, it is tempting to speculate that Sar-family proteins may regulate additional promoters by a variety of mechanisms including positive and negative influence on transcription arising from DNA bending, facilitating protein-DNA interaction of other factors, and perhaps combinatoric occupancy of Sar family binding sites. By downregulating SarS transcription via SarT, SarA may fine tune SarS levels which contribute to *tst* promoter regulation together with SarA [65]. Additional detailed studies will be required to resolve the details of this mechanism.

TSST-1 possesses an approximately 40 amino acid signal sequence and mature TSST-1 superantigen is predicted to be the 41–234 amino acid polypeptide. Interestingly, our western analysis detected strong induction of both the processed and unprocessed TSST-1 in DA140 compared with RN4282, where notably unprocessed TSST-1 precursor polypeptide was low or undetectable. Several scenarios might explain this observation. The loss of *sigB* could affect: 1) the proper expression of *spsB* encoding the Type II signal peptidase; 2) facilitate enhanced secretion via the sec-pathway; 3) impair extracellular proteolysis, or alternatively, 4) facilitate TSST-1 export via for example, non-sec dependent routes. The superantigenicity of the unprocessed TSST-1 polypeptide has to our knowledge, not been explored. We did not pursue the underlying cause of these observations further in this study.

Collectively, our results reported herein and integrated with additional published findings in Fig 6 show a complex network of regulation over *tst* and TSST-1 expression. Notably, the *tst* gene is not part of the core genome and resides embedded within one of several pathogenicity islands and we are unaware of exceptions to this observation [4, 12]. Recent work has shown these pathogenicity islands to be particularly efficient parasitic sequences dedicated to a lifestyle as mobilizable defective prophage [92]. Accessory genes, such as *tst* and other VF, may have been incorporated in them over the course of evolution. In this perspective, it is worthwhile considering the possibility that were *tst* not particularly well repressed by whatever means, its expression could interfere with pathogenicity island regulation. Prophage gene expression is strongly repressed by the islet-encoded *Stl* [93]. Derepression of Stl repressor would predictably lead to excision and the generation of a replicative form of the pathogenicity island, which could correspondingly rapidly increase the gene copy number and disrupting regulatory protein-DNA interaction stoichiometry. This would of course predict that factors such as bacteriophage infection, or genotoxic stress evoking an SOS response, including antibiotic stress such as that described for the induction of SOS by ciprofloxacin, could theoretically modulate *tst* production under these circumstances by transient gene amplification [93–97].

There are a number of physiological conditions that have been described that modulate *tst* expression and which do not yet have adequate underlying genetic regulatory explanation (Fig 6). This includes mild acid, magnesium, membrane-active agents, growth rate, pH, and oxygen. Aerobic conditions have been long thought to stimulate TSST-1 production, yet the nature of the oxygen sensor remains elusive. One candidate sensor is the SrrAB two-component system thought to modulate gene expression changes upon aerobic/anaerobic shift, although additional redox sensors controlling *tst* expression cannot be excluded [29, 30, 69].

Although further studies remain necessary to fully characterize and understand the regulatory patterns of TSST-1, current knowledge suggests that sporadic mutation in a few key negative regulators can profoundly affect and enhance *tst* expression. Mutations in *rsbU* or *sigB* were observed in laboratory strains, as in NCTC8325, but also in clinical isolates (K26, V8, Wood46) and were shown to strongly enhance exoprotein expression (notably Hla and SspA) [54, 98, 99].

## Supporting Information

S1 Fig Ponceau and Coomassie staining of strain supernatnats used in this study. Ponceau and Coomassie staining of strains used in the experiment of fig 3 (**A and B**) and Coomassie staining the various strains used in all other experiments **(C)**, in post-exponential growth phase (OD600 1.5 to 2). Carbonic anhydrase was added in each supernatant as a concentration and loading control. Carbonic anhydrase digestion in DA142 and DA156 lanes of panel C can be observed and is probably due to increased secretion of proteases in *sarA* mutants.
(TIF)

S2 Fig Graphics of normalized *C*_*T*_ values for qRT-PCR assays. Normalized cycle thresholds for n = 3 independent determinations for all figures presented in the text showing qRT-PCR data and prior to conversion to fold-change as described in Materials and Methods. The data are based upon the ct values presented in S1 Table. Each graphic displays Figure and corresponding probe used.
(DOCX)

S1 Table Normalized *C*_*T*_ values for all qRT-PCR assays. (DOCX)

## References

[1] Lowy FD. Staphylococcus aureus infections. N Engl J Med. 1998;339(8):520–32. Epub 1998/08/26. . 9709046 10.1056/NEJM199808203390806

[2] Gorwitz RJ, Kruszon-Moran D, McAllister SK, McQuillan G, McDougal LK, Fosheim GE, et al Changes in the prevalence of nasal colonization with Staphylococcus aureus in the United States, 2001–2004. J Infect Dis. 2008;197(9):1226–34. Epub 2008/04/22. 10.1086/533494 . 18422434

[3] Foster TJ. Colonization and infection of the human host by staphylococci: adhesion, survival and immune evasion. Vet Dermatol. 2009;20(5–6):456–70. Epub 2010/02/25. doi: VDE825 [pii] 10.1111/j.1365-3164.2009.00825.x . 20178484

[4] Novick RP. Mobile genetic elements and bacterial toxinoses: the superantigen-encoding pathogenicity islands of Staphylococcus aureus. Plasmid. 2003;49(2):93–105. Epub 2003/05/03. doi: S0147619X02001579 [pii]. . 12726763 10.1016/s0147-619x(02)00157-9

[5] Rooijakkers SH, van Kessel KP, van Strijp JA. Staphylococcal innate immune evasion. Trends Microbiol. 2005;13(12):596–601. Epub 2005/10/26. doi: S0966-842X(05)00280-5 [pii] 10.1016/j.tim.2005.10.002 . 16242332

[6] Spaan AN, Surewaard BG, Nijland R, van Strijp JA. Neutrophils versus Staphylococcus aureus: a biological tug of war. Annu Rev Microbiol. 2013;67:629–50. Epub 2013/07/10. 10.1146/annurev-micro-092412-155746 . 23834243

[7] van Wamel WJ, Rooijakkers SH, Ruyken M, van Kessel KP, van Strijp JA. The innate immune modulators staphylococcal complement inhibitor and chemotaxis inhibitory protein of Staphylococcus aureus are located on beta-hemolysin-converting bacteriophages. J Bacteriol. 2006;188(4):1310–5. Epub 2006/02/03. doi: 188/4/1310 [pii] 10.1128/JB.188.4.1310-1315.2006 16452413 PMC1367213

[8] Lappin E, Ferguson AJ. Gram-positive toxic shock syndromes. Lancet Infect Dis. 2009;9(5):281–90. Epub 2009/04/28. doi: S1473-3099(09)70066-0 [pii] 10.1016/S1473-3099(09)70066-0 . 19393958

[9] McCormick JK, Yarwood JM, Schlievert PM. Toxic shock syndrome and bacterial superantigens: an update. Annu Rev Microbiol. 2001;55:77–104. Epub 2001/09/07. doi: 10.1146/annurev.micro.55.1.77 55/1/77 [pii]. . 11544350 10.1146/annurev.micro.55.1.77

[10] Subedi A, Ubeda C, Adhikari RP, Penades JR, Novick RP. Sequence analysis reveals genetic exchanges and intraspecific spread of SaPI2, a pathogenicity island involved in menstrual toxic shock. Microbiology. 2007;153(Pt 10):3235–45. Epub 2007/10/02. doi: 153/10/3235 [pii] 10.1099/mic.0.2007/006932-0 . 17906123

[11] Kreiswirth BN, O'Reilly M, Novick RP. Genetic characterization and cloning of the toxic shock syndrome exotoxin. Surv Synth Pathol Res. 1984;3(1):73–82. Epub 1984/01/01. . 6438758

[12] Novick RP, Christie GE, Penades JR. The phage-related chromosomal islands of Gram-positive bacteria. Nat Rev Microbiol. 2010;8(8):541–51. Epub 2010/07/17. doi: nrmicro2393 [pii] 10.1038/nrmicro2393 . 20634809 PMC3522866

[13] Kreiswirth BN, Lofdahl S, Betley MJ, O'Reilly M, Schlievert PM, Bergdoll MS, et al The toxic shock syndrome exotoxin structural gene is not detectably transmitted by a prophage. Nature. 1983;305(5936):709–12. Epub 1983/10/20. . 6226876 10.1038/305709a0

[14] Parsonnet J, Hansmann MA, Delaney ML, Modern PA, Dubois AM, Wieland-Alter W, et al Prevalence of toxic shock syndrome toxin 1-producing Staphylococcus aureus and the presence of antibodies to this superantigen in menstruating women. J Clin Microbiol. 2005;43(9):4628–34. Epub 2005/09/08. doi: 43/9/4628 [pii] 10.1128/JCM.43.9.4628-4634.2005 16145118 PMC1234102

[15] Megevand C, Gervaix A, Heininger U, Berger C, Aebi C, Vaudaux B, et al Molecular epidemiology of the nasal colonization by methicillin-susceptible Staphylococcus aureus in Swiss children. Clin Microbiol Infect. 2010;16(9):1414–20. Epub 2009/10/23. 10.1111/j.1469-0691.2009.03090.x CLM3090 [pii]. . 19845693

[16] Lamy B, Laurent F, Gallon O, Doucet-Populaire F, Etienne J, Decousser JW. Antibacterial resistance, genes encoding toxins and genetic background among Staphylococcus aureus isolated from community-acquired skin and soft tissue infections in France: a national prospective survey. Eur J Clin Microbiol Infect Dis. 2012;31(6):1279–84. Epub 2011/10/15. 10.1007/s10096-011-1441-5 . 21997773

[17] Gaventa S, Reingold AL, Hightower AW, Broome CV, Schwartz B, Hoppe C, et al Active surveillance for toxic shock syndrome in the United States, 1986. Rev Infect Dis. 1989;11 Suppl 1:S28–34. Epub 1989/01/01. . 2928646 10.1093/clinids/11.supplement_1.s28

[18] Kreiswirth BN, Projan SJ, Schlievert PM, Novick RP. Toxic shock syndrome toxin 1 is encoded by a variable genetic element. Rev Infect Dis. 1989;11 Suppl 1:S83–8; discussion S8-9. Epub 1989/01/01. . 2564693 10.1093/clinids/11.supplement_1.s83

[19] Mir-Sanchis I, Martinez-Rubio R, Marti M, Chen J, Lasa I, Novick RP, et al Control of Staphylococcus aureus pathogenicity island excision. Mol Microbiol. 2012;85(5):833–45. Epub 2012/06/30. 10.1111/j.1365-2958.2012.08145.x . 22742067

[20] Lindsay JA, Ruzin A, Ross HF, Kurepina N, Novick RP. The gene for toxic shock toxin is carried by a family of mobile pathogenicity islands in Staphylococcus aureus. Mol Microbiol. 1998;29(2):527–43. Epub 1998/08/28. . 9720870 10.1046/j.1365-2958.1998.00947.x

[21] Horsburgh MJ, Aish JL, White IJ, Shaw L, Lithgow JK, Foster SJ. sigmaB modulates virulence determinant expression and stress resistance: characterization of a functional rsbU strain derived from Staphylococcus aureus 8325–4. J Bacteriol. 2002;184(19):5457–67. Epub 2002/09/10. 12218034 10.1128/JB.184.19.5457-5467.2002 PMC135357

[22] Chan PF, Foster SJ. The role of environmental factors in the regulation of virulence-determinant expression in Staphylococcus aureus 8325–4. Microbiology. 1998;144 (Pt 9):2469–79. Epub 1998/10/23. . 9782494 10.1099/00221287-144-9-2469

[23] Schlievert PM, Case LC, Nemeth KA, Davis CC, Sun Y, Qin W, et al Alpha and beta chains of hemoglobin inhibit production of Staphylococcus aureus exotoxins. Biochemistry. 2007;46(50):14349–58. Epub 2007/11/21. 10.1021/bi701202w 18020451 PMC2435367

[24] Kass EH. Magnesium and the pathogenesis of toxic shock syndrome. Rev Infect Dis. 1989;11 Suppl 1:S167–73; discussion S73-5. Epub 1989/01/01. . 2928633 10.1093/clinids/11.supplement_1.s167

[25] Seidl K, Bischoff M, Berger-Bachi B. CcpA mediates the catabolite repression of tst in Staphylococcus aureus. Infect Immun. 2008;76(11):5093–9. Epub 2008/08/20. doi: IAI.00724-08 [pii] 10.1128/IAI.00724-08 18710862 PMC2573359

[26] Vojtov N, Ross HF, Novick RP. Global repression of exotoxin synthesis by staphylococcal superantigens. Proc Natl Acad Sci U S A. 2002;99(15):10102–7. Epub 2002/07/12. 10.1073/pnas.152152499 152152499 [pii]. 12110733 PMC126631

[27] Yarwood JM, Schlievert PM. Oxygen and carbon dioxide regulation of toxic shock syndrome toxin 1 production by Staphylococcus aureus MN8. J Clin Microbiol. 2000;38(5):1797–803. Epub 2000/05/02. 10790102 10.1128/jcm.38.5.1797-1803.2000 PMC86591

[28] Novick RP. Autoinduction and signal transduction in the regulation of staphylococcal virulence. Mol Microbiol. 2003;48(6):1429–49. Epub 2003/06/07. doi: 3526 [pii]. . 12791129 10.1046/j.1365-2958.2003.03526.x

[29] Pragman AA, Yarwood JM, Tripp TJ, Schlievert PM. Characterization of virulence factor regulation by SrrAB, a two-component system in Staphylococcus aureus. J Bacteriol. 2004;186(8):2430–8. Epub 2004/04/03. 15060046 10.1128/JB.186.8.2430-2438.2004 PMC412142

[30] Pragman AA, Ji Y, Schlievert PM. Repression of Staphylococcus aureus SrrAB using inducible antisense srrA alters growth and virulence factor transcript levels. Biochemistry. 2007;46(1):314–21. Epub 2007/01/03. 10.1021/bi0603266 . 17198402

[31] Stevens DL, Ma Y, Salmi DB, McIndoo E, Wallace RJ, Bryant AE. Impact of antibiotics on expression of virulence-associated exotoxin genes in methicillin-sensitive and methicillin-resistant Staphylococcus aureus. J Infect Dis. 2007;195(2):202–11. Epub 2006/12/28. doi: JID36388 [pii] 10.1086/510396 . 17191165

[32] McNamara PJ, Syverson RE, Milligan-Myhre K, Frolova O, Schroeder S, Kidder J, et al Surfactants, aromatic and isoprenoid compounds, and fatty acid biosynthesis inhibitors suppress Staphylococcus aureus production of toxic shock syndrome toxin 1. Antimicrob Agents Chemother. 2009;53(5):1898–906. Epub 2009/02/19. 10.1128/AAC.01293-08 AAC.01293-08 [pii]. 19223628 PMC2681528

[33] Kimber I, Nookala S, Davis CC, Gerberick GF, Tucker H, Foertsch LM, et al Toxic shock syndrome: characterization of human immune responses to TSST-1 and evidence for sensitivity thresholds. Toxicol Sci. 2013;134(1):49–63. Epub 2013/05/04. 10.1093/toxsci/kft099 kft099 [pii]. . 23640863

[34] Li J, Wang W, Xu SX, Magarvey NA, McCormick JK. Lactobacillus reuteri-produced cyclic dipeptides quench agr-mediated expression of toxic shock syndrome toxin-1 in staphylococci. Proc Natl Acad Sci U S A. 2011;108(8):3360–5. Epub 2011/02/02. doi: 1017431108 [pii] 10.1073/pnas.1017431108 21282650 PMC3044419

[35] Nagao M, Okamoto A, Yamada K, Hasegawa T, Hasegawa Y, Ohta M. Variations in amount of TSST-1 produced by clinical methicillin resistant Staphylococcus aureus (MRSA) isolates and allelic variation in accessory gene regulator (agr) locus. BMC Microbiol. 2009;9:52 Epub 2009/03/11. 10.1186/1471-2180-9-52 1471-2180-9-52 [pii]. 19272162 PMC2667389

[36] Bronner S, Monteil H, Prevost G. Regulation of virulence determinants in Staphylococcus aureus: complexity and applications. FEMS Microbiol Rev. 2004;28(2):183–200. Epub 2004/04/28. 10.1016/j.femsre.2003.09.003 S0168644503000925 [pii]. . 15109784

[37] Cheung AL, Bayer AS, Zhang G, Gresham H, Xiong YQ. Regulation of virulence determinants in vitro and in vivo in Staphylococcus aureus. FEMS Immunol Med Microbiol. 2004;40(1):1–9. Epub 2004/01/22. doi: S0928824403003092 [pii]. . 14734180 10.1016/S0928-8244(03)00309-2

[38] Recsei P, Kreiswirth B, O'Reilly M, Schlievert P, Gruss A, Novick RP. Regulation of exoprotein gene expression in Staphylococcus aureus by agar. Mol Gen Genet. 1986;202(1):58–61. Epub 1986/01/01. . 3007938 10.1007/BF00330517

[39] Chan PF, Foster SJ. Role of SarA in virulence determinant production and environmental signal transduction in Staphylococcus aureus. J Bacteriol. 1998;180(23):6232–41. Epub 1998/11/26. 9829932 10.1128/jb.180.23.6232-6241.1998 PMC107708

[40] Andrey DO, Renzoni A, Monod A, Lew DP, Cheung AL, Kelley WL. Control of the Staphylococcus aureus toxic shock tst promoter by the global regulator SarA. J Bacteriol. 2010;192(22):6077–85. Epub 2010/09/28. doi: JB.00146-10 [pii] 10.1128/JB.00146-10 20870770 PMC2976458

[41] McNamara PJ, Milligan-Monroe KC, Khalili S, Proctor RA. Identification, cloning, and initial characterization of rot, a locus encoding a regulator of virulence factor expression in Staphylococcus aureus. J Bacteriol. 2000;182(11):3197–203. Epub 2000/05/16. 10809700 10.1128/jb.182.11.3197-3203.2000 PMC94507

[42] Said-Salim B, Dunman PM, McAleese FM, Macapagal D, Murphy E, McNamara PJ, et al Global regulation of Staphylococcus aureus genes by Rot. J Bacteriol. 2003;185(2):610–9. Epub 2003/01/04. 12511508 10.1128/JB.185.2.610-619.2003 PMC145333

[43] Felden B, Vandenesch F, Bouloc P, Romby P. The Staphylococcus aureus RNome and its commitment to virulence. PLoS Pathog. 2011;7(3):e1002006 Epub 2011/03/23. 10.1371/journal.ppat.1002006 21423670 PMC3053349

[44] Leiba J, Hartmann T, Cluzel ME, Cohen-Gonsaud M, Delolme F, Bischoff M, et al A novel mode of regulation of the Staphylococcus aureus catabolite control protein A (CcpA) mediated by Stk1 protein phosphorylation. J Biol Chem. 2012;287(52):43607–19. Epub 2012/11/08. 10.1074/jbc.M112.418913 M112.418913 [pii]. 23132867 PMC3527947

[45] Ohlsen K, Donat S. The impact of serine/threonine phosphorylation in Staphylococcus aureus. Int J Med Microbiol. 2010;300(2–3):137–41. Epub 2009/09/29. doi: S1438-4221(09)00109-X [pii] 10.1016/j.ijmm.2009.08.016 . 19783479

[46] Yarwood JM, McCormick JK, Schlievert PM. Identification of a novel two-component regulatory system that acts in global regulation of virulence factors of Staphylococcus aureus. J Bacteriol. 2001;183(4):1113–23. Epub 2001/02/07. 10.1128/JB.183.4.1113-1123.2001 11157922 PMC94983

[47] Tu Quoc PH, Genevaux P, Pajunen M, Savilahti H, Georgopoulos C, Schrenzel J, et al Isolation and characterization of biofilm formation-defective mutants of Staphylococcus aureus. Infect Immun. 2007;75(3):1079–88. Epub 2006/12/13. doi: IAI.01143-06 [pii] 10.1128/IAI.01143-06 17158901 PMC1828571

[48] Bischoff M, Dunman P, Kormanec J, Macapagal D, Murphy E, Mounts W, et al Microarray-based analysis of the Staphylococcus aureus sigmaB regulon. J Bacteriol. 2004;186(13):4085–99. Epub 2004/06/19. 10.1128/JB.186.13.4085-4099.2004 186/13/4085 [pii]. 15205410 PMC421609

[49] Oscarsson J, Harlos C, Arvidson S. Regulatory role of proteins binding to the spa (protein A) and sarS (staphylococcal accessory regulator) promoter regions in Staphylococcus aureus NTCC 8325–4. Int J Med Microbiol. 2005;295(4):253–66. Epub 2005/09/01. . 16128400 10.1016/j.ijmm.2005.05.003

[50] Renzoni A, Kelley WL, Barras C, Monod A, Huggler E, Francois P, et al Identification by genomic and genetic analysis of two new genes playing a key role in intermediate glycopeptide resistance in Staphylococcus aureus. Antimicrob Agents Chemother. 2009;53(3):903–11. Epub 2008/12/24. doi: AAC.01287-08 [pii] 10.1128/AAC.01287-08 19104009 PMC2650575

[51] Renzoni A, Francois P, Li D, Kelley WL, Lew DP, Vaudaux P, et al Modulation of fibronectin adhesins and other virulence factors in a teicoplanin-resistant derivative of methicillin-resistant Staphylococcus aureus. Antimicrob Agents Chemother. 2004;48(8):2958–65. Epub 2004/07/27. 10.1128/AAC.48.8.2958-2965.2004 48/8/2958 [pii]. 15273106 PMC478536

[52] Vaudaux P, Francois P, Bisognano C, Kelley WL, Lew DP, Schrenzel J, et al Increased expression of clumping factor and fibronectin-binding proteins by hemB mutants of Staphylococcus aureus expressing small colony variant phenotypes. Infect Immun. 2002;70(10):5428–37. Epub 2002/09/14. 12228267 10.1128/IAI.70.10.5428-5437.2002 PMC128368

[53] Kullik II, Giachino P. The alternative sigma factor sigmaB in Staphylococcus aureus: regulation of the sigB operon in response to growth phase and heat shock. Arch Microbiol. 1997;167(2/3):151–9. Epub 1997/03/07. doi: 71670151.203 [pii]. . 9042755 10.1007/s002030050428

[54] Herbert S, Ziebandt AK, Ohlsen K, Schafer T, Hecker M, Albrecht D, et al Repair of global regulators in Staphylococcus aureus 8325 and comparative analysis with other clinical isolates. Infect Immun. 2010;78(6):2877–89. Epub 2010/03/10. doi: IAI.00088-10 [pii] 10.1128/IAI.00088-10 20212089 PMC2876537

[55] Giachino P, Engelmann S, Bischoff M. Sigma(B) activity depends on RsbU in Staphylococcus aureus. J Bacteriol. 2001;183(6):1843–52. Epub 2001/02/27. 10.1128/JB.183.6.1843-1852.2001 11222581 PMC95078

[56] Haldenwang WG. The sigma factors of Bacillus subtilis. Microbiol Rev. 1995;59(1):1–30. Epub 1995/03/01. 7708009 10.1128/mr.59.1.1-30.1995 PMC239352

[57] Gertz S, Engelmann S, Schmid R, Ziebandt AK, Tischer K, Scharf C, et al Characterization of the sigma(B) regulon in Staphylococcus aureus. J Bacteriol. 2000;182(24):6983–91. Epub 2000/11/28. 11092859 10.1128/jb.182.24.6983-6991.2000 PMC94824

[58] Bischoff M, Entenza JM, Giachino P. Influence of a functional sigB operon on the global regulators sar and agr in Staphylococcus aureus. J Bacteriol. 2001;183(17):5171–9. Epub 2001/08/08. 11489871 10.1128/JB.183.17.5171-5179.2001 PMC95394

[59] Tegmark K, Karlsson A, Arvidson S. Identification and characterization of SarH1, a new global regulator of virulence gene expression in Staphylococcus aureus. Mol Microbiol. 2000;37(2):398–409. Epub 2000/08/10. doi: mmi2003 [pii]. . 10931334 10.1046/j.1365-2958.2000.02003.x

[60] Geisinger E, Adhikari RP, Jin R, Ross HF, Novick RP. Inhibition of rot translation by RNAIII, a key feature of agr function. Mol Microbiol. 2006;61(4):1038–48. Epub 2006/08/02. doi: MMI5292 [pii] 10.1111/j.1365-2958.2006.05292.x . 16879652

[61] Boisset S, Geissmann T, Huntzinger E, Fechter P, Bendridi N, Possedko M, et al Staphylococcus aureus RNAIII coordinately represses the synthesis of virulence factors and the transcription regulator Rot by an antisense mechanism. Genes Dev. 2007;21(11):1353–66. Epub 2007/06/05. doi: 21/11/1353 [pii] 10.1101/gad.423507 17545468 PMC1877748

[62] Benson MA, Lilo S, Nygaard T, Voyich JM, Torres VJ. Rot and SaeRS cooperate to activate expression of the staphylococcal superantigen-like exoproteins. J Bacteriol. 2012;194(16):4355–65. Epub 2012/06/12. 10.1128/JB.00706-12JB.00706-12 [pii]. 22685286 PMC3416255

[63] Cheung AL, Schmidt K, Bateman B, Manna AC. SarS, a SarA homolog repressible by agr, is an activator of protein A synthesis in Staphylococcus aureus. Infect Immun. 2001;69(4):2448–55. Epub 2001/03/20. 10.1128/IAI.69.4.2448-2455.2001 11254606 PMC98178

[64] Oscarsson J, Kanth A, Tegmark-Wisell K, Arvidson S. SarA is a repressor of hla (alpha-hemolysin) transcription in Staphylococcus aureus: its apparent role as an activator of hla in the prototype strain NCTC 8325 depends on reduced expression of sarS. J Bacteriol. 2006;188(24):8526–33. Epub 2006/10/03. doi: JB.00866-06 [pii] 10.1128/JB.00866-06 17012389 PMC1698246

[65] Schmidt KA, Manna AC, Cheung AL. SarT influences sarS expression in Staphylococcus aureus. Infect Immun. 2003;71(9):5139–48. Epub 2003/08/23. 12933857 10.1128/IAI.71.9.5139-5148.2003 PMC187355

[66] Cheung AL, Nishina KA, Trotonda MP, Tamber S. The SarA protein family of Staphylococcus aureus. Int J Biochem Cell Biol. 2008;40(3):355–61. Epub 2007/12/18. doi: S1357-2725(07)00359-7 [pii] 10.1016/j.biocel.2007.10.032 18083623 PMC2274939

[67] Lindsay JA, Moore CE, Day NP, Peacock SJ, Witney AA, Stabler RA, et al Microarrays reveal that each of the ten dominant lineages of Staphylococcus aureus has a unique combination of surface-associated and regulatory genes. J Bacteriol. 2006;188(2):669–76. Epub 2005/12/31. doi: 188/2/669 [pii] 10.1128/JB.188.2.669-676.2006 16385056 PMC1347281

[68] Karlsson A, Saravia-Otten P, Tegmark K, Morfeldt E, Arvidson S. Decreased amounts of cell wall-associated protein A and fibronectin-binding proteins in Staphylococcus aureus sarA mutants due to up-regulation of extracellular proteases. Infect Immun. 2001;69(8):4742–8. Epub 2001/07/12. 10.1128/IAI.69.8.4742-4748.2001 11447146 PMC98560

[69] Somerville GA, Proctor RA. At the crossroads of bacterial metabolism and virulence factor synthesis in Staphylococci. Microbiol Mol Biol Rev. 2009;73(2):233–48. Epub 2009/06/03. doi: 73/2/233 [pii] 10.1128/MMBR.00005-09 19487727 PMC2698418

[70] Burnside K, Lembo A, de Los Reyes M, Iliuk A, Binhtran NT, Connelly JE, et al Regulation of hemolysin expression and virulence of Staphylococcus aureus by a serine/threonine kinase and phosphatase. PLoS One. 2010;5(6):e11071 Epub 2010/06/17. 10.1371/journal.pone.0011071 20552019 PMC2884019

[71] Chen PR, Nishida S, Poor CB, Cheng A, Bae T, Kuechenmeister L, et al A new oxidative sensing and regulation pathway mediated by the MgrA homologue SarZ in Staphylococcus aureus. Mol Microbiol. 2009;71(1):198–211. Epub 2008/11/15. doi: MMI6518 [pii] 10.1111/j.1365-2958.2008.06518.x 19007410 PMC2698432

[72] Didier JP, Cozzone AJ, Duclos B. Phosphorylation of the virulence regulator SarA modulates its ability to bind DNA in Staphylococcus aureus. FEMS Microbiol Lett. 2010;306(1):30–6. Epub 2010/03/27. doi: FML1930 [pii] 10.1111/j.1574-6968.2010.01930.x . 20337713

[73] Manna AC, Bayer MG, Cheung AL. Transcriptional analysis of different promoters in the sar locus in Staphylococcus aureus. J Bacteriol. 1998;180(15):3828–36. Epub 1998/07/31. 9683479 10.1128/jb.180.15.3828-3836.1998 PMC107366

[74] Meier S, Goerke C, Wolz C, Seidl K, Homerova D, Schulthess B, et al sigmaB and the sigmaB-dependent arlRS and yabJ-spoVG loci affect capsule formation in Staphylococcus aureus. Infect Immun. 2007;75(9):4562–71. Epub 2007/07/20. doi: IAI.00392-07 [pii] 10.1128/IAI.00392-07 17635871 PMC1951174

[75] Schulthess B, Bloes DA, Francois P, Girard M, Schrenzel J, Bischoff M, et al The sigmaB-dependent yabJ-spoVG operon is involved in the regulation of extracellular nuclease, lipase, and protease expression in Staphylococcus aureus. J Bacteriol. 2011;193(18):4954–62. Epub 2011/07/05. 10.1128/JB.05362-11 JB.05362-11 [pii]. 21725011 PMC3165683

[76] Liang X, Zheng L, Landwehr C, Lunsford D, Holmes D, Ji Y. Global regulation of gene expression by ArlRS, a two-component signal transduction regulatory system of Staphylococcus aureus. J Bacteriol. 2005;187(15):5486–92. Epub 2005/07/21. doi: 187/15/5486-a [pii] 10.1128/JB.187.15.5486-5492.2005 16030243 PMC1196029

[77] Schulthess B, Bloes DA, Berger-Bachi B. Opposing roles of sigmaB and sigmaB-controlled SpoVG in the global regulation of esxA in Staphylococcus aureus. BMC Microbiol. 2012;12:17 Epub 2012/01/26. [pii]. 22272815 10.1186/1471-2180-12-17 PMC3313859

[78] Geissmann T, Chevalier C, Cros MJ, Boisset S, Fechter P, Noirot C, et al A search for small noncoding RNAs in Staphylococcus aureus reveals a conserved sequence motif for regulation. Nucleic Acids Res. 2009;37(21):7239–57. Epub 2009/09/30. doi: gkp668 [pii] 10.1093/nar/gkp668 19786493 PMC2790875

[79] Nielsen JS, Christiansen MH, Bonde M, Gottschalk S, Frees D, Thomsen LE, et al Searching for small sigmaB-regulated genes in Staphylococcus aureus. Arch Microbiol. 2011;193(1):23–34. Epub 2010/10/28. 10.1007/s00203-010-0641-1 . 20978742

[80] Koch G, Yepes A, Forstner KU, Wermser C, Stengel ST, Modamio J, et al Evolution of resistance to a last-resort antibiotic in Staphylococcus aureus via bacterial competition. Cell. 2014;158(5):1060–71. 10.1016/j.cell.2014.06.046 25171407 PMC4163622

[81] Cheung AL, Koomey JM, Butler CA, Projan SJ, Fischetti VA. Regulation of exoprotein expression in Staphylococcus aureus by a locus (sar) distinct from agr. Proc Natl Acad Sci U S A. 1992;89(14):6462–6. Epub 1992/07/15. 1321441 10.1073/pnas.89.14.6462 PMC49521

[82] Heinrichs JH, Bayer MG, Cheung AL. Characterization of the sar locus and its interaction with agr in Staphylococcus aureus. J Bacteriol. 1996;178(2):418–23. Epub 1996/01/01. 8550461 10.1128/jb.178.2.418-423.1996 PMC177673

[83] Blevins JS, Beenken KE, Elasri MO, Hurlburt BK, Smeltzer MS. Strain-dependent differences in the regulatory roles of sarA and agr in Staphylococcus aureus. Infect Immun. 2002;70(2):470–80. Epub 2002/01/18. 11796572 10.1128/IAI.70.2.470-480.2002 PMC127691

[84] Zielinska AK, Beenken KE, Joo HS, Mrak LN, Griffin LM, Luong TT, et al Defining the Strain-Dependent Impact of the Staphylococcal Accessory Regulator (sarA) on the Alpha-Toxin Phenotype of Staphylococcus aureus. J Bacteriol. 2011;193(12):2948–58. Epub 2011/04/12. doi: JB.01517-10 [pii] 10.1128/JB.01517-10 . 21478342 PMC3133183

[85] Cheung AL, Ying P. Regulation of alpha- and beta-hemolysins by the sar locus of Staphylococcus aureus. J Bacteriol. 1994;176(3):580–5. Epub 1994/02/01. 7507919 10.1128/jb.176.3.580-585.1994 PMC205093

[86] Chien Y, Manna AC, Projan SJ, Cheung AL. SarA, a global regulator of virulence determinants in Staphylococcus aureus, binds to a conserved motif essential for sar-dependent gene regulation. J Biol Chem. 1999;274(52):37169–76. Epub 1999/12/22. . 10601279 10.1074/jbc.274.52.37169

[87] Jelsbak L, Hemmingsen L, Donat S, Ohlsen K, Boye K, Westh H, et al Growth phase-dependent regulation of the global virulence regulator Rot in clinical isolates of Staphylococcus aureus. Int J Med Microbiol. 2010;300(4):229–36. Epub 2009/08/12. doi: S1438-4221(09)00058-7 [pii] 10.1016/j.ijmm.2009.07.003 . 19665927

[88] McCallum N, Bischoff M, Maki H, Wada A, Berger-Bachi B. TcaR, a putative MarR-like regulator of sarS expression. J Bacteriol. 2004;186(10):2966–72. Epub 2004/05/06. 15126456 10.1128/JB.186.10.2966-2972.2004 PMC400606

[89] Baek KT, Frees D, Renzoni A, Barras C, Rodriguez N, Manzano C, et al Genetic variation in the Staphylococcus aureus 8325 strain lineage revealed by whole-genome sequencing. PLoS One. 2013;8(9):e77122 10.1371/journal.pone.0077122 24098817 PMC3786944

[90] Ballal A, Manna AC. Expression of the sarA family of genes in different strains of Staphylococcus aureus. Microbiology. 2009;155(Pt 7):2342–52. Epub 2009/04/25. doi: mic.0.027417–0 [pii] 10.1099/mic.0.027417-0 19389785 PMC2888119

[91] Reyes D, Andrey DO, Monod A, Kelley WL, Zhang G, Cheung AL. Coordinated regulation by AgrA, SarA, and SarR to control agr expression in Staphylococcus aureus. J Bacteriol. 2011;193(21):6020–31. Epub 2011/09/13. 10.1128/JB.05436-11 JB.05436-11 [pii]. 21908676 PMC3194896

[92] Ram G, Chen J, Kumar K, Ross HF, Ubeda C, Damle PK, et al Staphylococcal pathogenicity island interference with helper phage reproduction is a paradigm of molecular parasitism. Proc Natl Acad Sci U S A. 2012;109(40):16300–5. Epub 2012/09/20. 10.1073/pnas.1204615109 1204615109 [pii]. 22991467 PMC3479557

[93] Ubeda C, Maiques E, Barry P, Matthews A, Tormo MA, Lasa I, et al SaPI mutations affecting replication and transfer and enabling autonomous replication in the absence of helper phage. Mol Microbiol. 2008;67(3):493–503. Epub 2007/12/19. doi: MMI6027 [pii] 10.1111/j.1365-2958.2007.06027.x . 18086210

[94] Bisognano C, Kelley WL, Estoppey T, Francois P, Schrenzel J, Li D, et al A recA-LexA-dependent pathway mediates ciprofloxacin-induced fibronectin binding in Staphylococcus aureus. J Biol Chem. 2004;279(10):9064–71. Epub 2003/12/31. 10.1074/jbc.M309836200 M309836200 [pii]. . 14699158

[95] Ubeda C, Maiques E, Knecht E, Lasa I, Novick RP, Penades JR. Antibiotic-induced SOS response promotes horizontal dissemination of pathogenicity island-encoded virulence factors in staphylococci. Mol Microbiol. 2005;56(3):836–44. Epub 2005/04/12. doi: MMI4584 [pii] 10.1111/j.1365-2958.2005.04584.x . 15819636

[96] Maiques E, Ubeda C, Campoy S, Salvador N, Lasa I, Novick RP, et al beta-lactam antibiotics induce the SOS response and horizontal transfer of virulence factors in Staphylococcus aureus. J Bacteriol. 2006;188(7):2726–9. Epub 2006/03/21. doi: 188/7/2726 [pii] 10.1128/JB.188.7.2726-2729.2006 16547063 PMC1428414

[97] Tormo-Mas MA, Mir I, Shrestha A, Tallent SM, Campoy S, Lasa I, et al Moonlighting bacteriophage proteins derepress staphylococcal pathogenicity islands. Nature. 2010;465(7299):779–82. Epub 2010/05/18. doi: nature09065 [pii] 10.1038/nature09065 . 20473284 PMC3518041

[98] Karlsson-Kanth A, Tegmark-Wisell K, Arvidson S, Oscarsson J. Natural human isolates of Staphylococcus aureus selected for high production of proteases and alpha-hemolysin are sigmaB deficient. Int J Med Microbiol. 2006;296(4–5):229–36. Epub 2006/03/15. doi: S1438-4221(06)00075-0 [pii] 10.1016/j.ijmm.2006.01.067 . 16530010

[99] Cheung AL, Chien YT, Bayer AS. Hyperproduction of alpha-hemolysin in a sigB mutant is associated with elevated SarA expression in Staphylococcus aureus. Infect Immun. 1999;67(3):1331–7. Epub 1999/02/20. 10024579 10.1128/iai.67.3.1331-1337.1999 PMC96465

[100] Schlievert PM, Merriman JA, Salgado-Pabon W, Mueller EA, Spaulding AR, Vu BG, et al Menaquinone analogs inhibit growth of bacterial pathogens. Antimicrob Agents Chemother. 2013;57(11):5432–7. Epub 2013/08/21. 10.1128/AAC.01279-13 [pii]. 23959313 PMC3811306

[101] Sun F, Li C, Jeong D, Sohn C, He C, Bae T. In the Staphylococcus aureus two-component system sae, the response regulator SaeR binds to a direct repeat sequence and DNA binding requires phosphorylation by the sensor kinase SaeS. J Bacteriol. 2010;192(8):2111–27. Epub 2010/02/23. doi: JB.01524-09 [pii] 10.1128/JB.01524-09 20172998 PMC2849438

[102] Weinrick B, Dunman PM, McAleese F, Murphy E, Projan SJ, Fang Y, et al Effect of mild acid on gene expression in Staphylococcus aureus. J Bacteriol. 2004;186(24):8407–23. Epub 2004/12/04. doi: 186/24/8407 [pii] 10.1128/JB.186.24.8407-8423.2004 15576791 PMC532443

[103] Sarafian SK, Morse SA. Environmental factors affecting toxic shock syndrome toxin-1 (TSST-1) synthesis. J Med Microbiol. 1987;24(1):75–81. Epub 1987/08/01. . 3612745 10.1099/00222615-24-1-75

[104] Schlievert PM, Kelly JA. Clindamycin-induced suppression of toxic-shock syndrome—associated exotoxin production. J Infect Dis. 1984;149(3):471 Epub 1984/03/01. . 6715902 10.1093/infdis/149.3.471

[105] Kiyota H, Kendrick MI, Kass EH. Influence of magnesium concentration on production of exoprotein and beta-lactamase by Staphylococcus aureus and Staphylococcus hemolyticus. J Infect Dis. 1989;160(6):1061–3. Epub 1989/12/01. . 2584753 10.1093/infdis/160.6.1061

[106] Taylor D, Holland KT. Production of toxic shock syndrome toxin 1 by Staphylococcus aureus under aerobic and anaerobic conditions and the effect of magnesium ion limitation. Rev Infect Dis. 1989;11 Suppl 1:S151–6. Epub 1989/01/01. . 2928631 10.1093/clinids/11.supplement_1.s151

[107] Mills JT, Dodel AW, Kass EH. Regulation of staphylococcal toxic shock syndrome toxin-1 and total exoprotein production by magnesium ion. Infect Immun. 1986;53(3):663–70. Epub 1986/09/01. 3527988 10.1128/iai.53.3.663-670.1986 PMC260845

