# Supplementary figures and images for: Impact of the Regulators SigB, Rot, SarA and sarS on the Toxic Shock Tst Promoter and TSST-1 Expression in Staphylococcus aureus
Source: PLoS One. 2015 Aug 14;10(8):e0135579. doi: 10.1371/journal.pone.0135579 (PMC4537247; doi:10.1371/journal.pone.0135579)

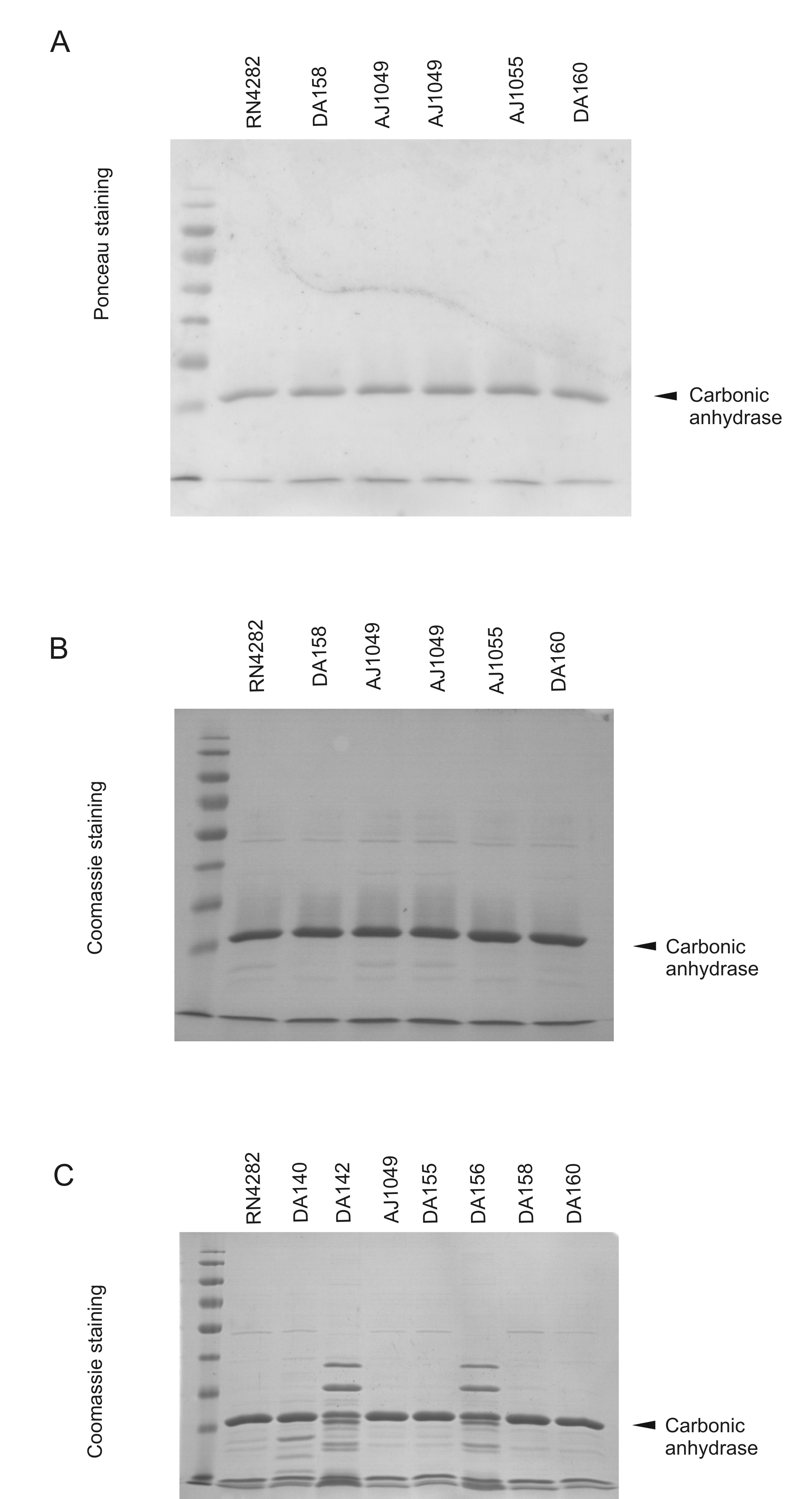

Supplement: S1 Fig — Ponceau and Coomassie staining of strains used in the experiment of fig 3 (A and B) and Coomassie staining the various strains used in all other experiments (C), in post-exponential growth phase (OD600 1.5 to 2). Carbonic anhydrase was added in each supernatant as a concentration and loading control. Carbonic anhydrase digestion in DA142 and DA156 lanes of panel C can be observed and is probably due to increased secretion of proteases in sarA mutants. (TIF) [file pone.0135579.s001.tif]
