# Supplementary material for: Impact of the Regulators SigB, Rot, SarA and sarS on the Toxic Shock Tst Promoter and TSST-1 Expression in Staphylococcus aureus
Source: PLoS One. 2015 Aug 14;10(8):e0135579. doi: 10.1371/journal.pone.0135579 (PMC4537247; doi:10.1371/journal.pone.0135579)
Supplement: S1 Table — (DOCX) [file pone.0135579.s003.docx]

| **Supplementary Table 1. Normalized ct values for all qRT-PCR assays.** | | | | | |
| --- | --- | --- | --- | --- | --- |
|  | CT values |  |  |  |  |
|  | #1 | #2 | #3 | mean | SD |
| Fig1B tst |  |  |  |  |  |
| RN4282 | 18.15 | 18.22 | 18.2 | 18.19 | 0.036055513 |
| DA140 | 13.16 | 13.77 | 14.68 | 13.87 | 0.764918296 |
| DA141 | 15.74 | 16.28 | 16.24 | 16.08666667 | 0.300887576 |
|  |  |  |  |  |  |
| Fig2A RNAIII | |  |  |  |  |
| RN4282 | 17.1 | 17.33 | 17.25 | 17.22666667 | 0.116761866 |
| DA140 | 16.37 | 16.23 | 16.55 | 16.38333333 | 0.160416126 |
| DA141 | 16.99 | 18.26 | 17.91 | 17.72 | 0.65597256 |
|  |  |  |  |  |  |
| Fig2B sarA |  |  |  |  |  |
| RN4282 | 22.98 | 22.23 | 23.63 | 22.94666667 | 0.700594985 |
| DA140 | 25.16 | 25.91 | 25.89 | 25.65333333 | 0.427356214 |
| DA141 | 24.18 | 23.63 | 23.79 | 23.86666667 | 0.282901632 |
|  |  |  |  |  |  |
| Fig2C sarS |  |  |  |  |  |
| RN4282 | 19.62 | 19.47 | 19.41 | 19.5 | 0.108166538 |
| DA140 | 18.99 | 19.77 | 18.76 | 19.17333333 | 0.529370696 |
| DA141 | 19.37 | 18.99 | 19.15 | 19.17 | 0.19078784 |
|  |  |  |  |  |  |
| Fig4A tst |  |  |  |  |  |
| RN4282 | 20.32 | 20.63 | 18.95 | 19.96666667 | 0.893998509 |
| DA142 | 18.42 | 18.93 | 17.08 | 18.14333333 | 0.955527777 |
| AJ1062 | 23.88 | 22.44 | 23.57 | 23.29666667 | 0.757913803 |
| Fig4C tst |  |  |  |  |  |
| RN4282 | 18.44 | 18.51 | 18.5 | 18.48333333 | 0.037859389 |
| DA155 | 17.07 | 16.94 | 17.1 | 17.03666667 | 0.085049005 |
| DA156 | 16.19 | 15.8 | 16.9 | 16.29666667 | 0.557703625 |
|  |  |  |  |  |  |
|  |  |  |  |  |  |
| **Fig3 *tst*** |  |  |  |  |  |
| RN4282 | 20.1 | 20.78 | 20.52 | 20.46666667 | 0.480832611 |
| DA158 | 22.75 | 23.64 | 22.95 | 23.11333333 | 0.629325035 |
| DA160 | 19.45 | 20.18 | 19.9 | 19.84333333 | 0.51618795 |
|  |  |  |  |  |  |
| RN4282 | 18.79 | 18.52 | 19.04 | 18.783333 | 0.190918831 |
| AJ1055 | 23.80 | 22.63 | 23.20 | 23.21 | 0.585064099 |
| AJ1049 | 19.64 | 19.59 | 19.30 | 19.51 | 0.183575598 |

|  | |  | |  |  | |  | |  | |
| --- | --- | --- | --- | --- | --- | --- | --- | --- | --- | --- |
|  | |  | |  |  | |  | |  | |
|  |  | |  | | |  | |  | |  |
|  |  | | RN4282 | | | DA140 | | DA141 | |  |
|  | Mean | | 18.19 | | | 13.87 | | 16.09 | |  |
|  | SD | | 0.04 | | | 0.76 | | 0.30 | |  |
|  |  | |  | | |  | |  | |  |
|  |  | |  | | |  | |  | |  |
|  |  | | RN4282 | | | DA140 | | DA141 | |  |
|  | Mean | | 17.23 | | | 16.38 | | 17.72 | |  |
|  | SD | | 0.12 | | | 0.16 | | 0.66 | |  |
|  |  | |  | | |  | |  | |  |
|  |  | |  | | |  | |  | |  |
|  |  | | RN4282 | | | DA140 | | DA141 | |  |
|  | Mean | | 22.95 | | | 25.65 | | 23.87 | |  |
|  | SD | | 0.70 | | | 0.43 | | 0.28 | |  |
|  |  | |  | | |  | |  | |  |
|  |  | |  | | |  | |  | |  |
|  |  | | RN4282 | | | DA140 | | DA141 | |  |
|  | Mean | | 19.50 | | | 19.17 | | 19.17 | |  |
|  | SD | | 0.11 | | | 0.53 | | 0.19 | |  |
|  |  | |  | | |  | |  | |  |
|  |  | |  | | |  | |  | |  |
|  |  | | RN4282 | | | DA142 | | AJ1062 | |  |
|  | Mean | | 19.96666667 | | | 18.14333333 | | 23.29666667 | |  |
|  | SD | | 0.893998509 | | | 0.955527777 | | 0.757913803 | |  |
|  |  | |  | | |  | |  | |  |
|  |  | | RN4282 | | | DA155 | | DA156 | |  |
|  | Mean | | 18.48333333 | | | 17.03666667 | | 16.29666667 | |  |
|  | SD | | 0.037859389 | | | 0.085049005 | | 0.557703625 | |  |
|  |  | |  | | |  | |  | |  |
|  |  | |  | | |  | |  | |  |
|  |  | |  | | |  | |  | |  |
|  |  | | RN4282 | | | DA158 | | DA160 | |  |
|  | Mean | | 20.46666667 | | | 23.11333333 | | 19.84333333 | |  |
|  | SD | | 0.480832611 | | | 0.629325035 | | 0.51618795 | |  |
|  |  | |  | | |  | |  | |  |
|  |  | | RN4282 | | | AJ1055 | | AJ1049 | |  |
|  | Mean | | 18.783333 | | | 23.21 | | 19.51 | |  |
|  | SD | | 0.190918831 | | | 0.585064099 | | 0.183575598 | |  |
|  |  | |  | | |  | |  | |  |
